# Engineering heat tolerance in potato by temperature‐dependent expression of a specific allele of *HEAT‐SHOCK COGNATE 70*

**DOI:** 10.1111/pbi.12760

**Published:** 2017-06-20

**Authors:** Almudena Trapero‐Mozos, Wayne L. Morris, Laurence J. M. Ducreux, Karen McLean, Jennifer Stephens, Lesley Torrance, Glenn J. Bryan, Robert D. Hancock, Mark A. Taylor

**Affiliations:** ^1^ School of Biology University of St Andrews St Andrews Fife UK; ^2^ Cell and Molecular Sciences The James Hutton Institute Dundee UK

**Keywords:** *HEAT‐SHOCK COGNATE 70*, heat tolerance, potato, promoter, quantitative trait locus, yield

## Abstract

For many commercial potato cultivars, tuber yield is optimal at average daytime temperatures in the range of 14–22 °C. Further rises in ambient temperature can reduce or completely inhibit potato tuber production, with damaging consequences for both producer and consumer. The aim of this study was to use a genetic screen based on a model tuberization assay to identify quantitative trait loci (QTL) associated with enhanced tuber yield. A candidate gene encoding HSc70 was identified within one of the three QTL intervals associated with elevated yield in a Phureja–Tuberosum hybrid diploid potato population (06H1). A particular *HSc70* allelic variant was linked to elevated yield in the 06H1 progeny. Expression of this allelic variant was much higher than other alleles, particularly on exposure to moderately elevated temperature. Transient expression of this allele in *Nicotiana benthamiana* resulted in significantly enhanced tolerance to elevated temperature. An TA repeat element was present in the promoter of this allele, but not in other *HSc70* alleles identified in the population. Expression of the *HSc70* allelic variant under its native promoter in the potato cultivar Desiree resulted in enhanced *HSc70* expression at elevated temperature. This was reflected in greater tolerance to heat stress as determined by improved yield under moderately elevated temperature in a model nodal cutting tuberization system and in plants grown from stem cuttings. Our results identify *HSc70* expression level as a significant factor influencing yield stability under moderately elevated temperature and identify specific allelic variants of *HSc70* for the induction of thermotolerance via conventional introgression or molecular breeding approaches.

## Introduction

Potato is the third most important food crop in the world after rice and wheat. More than a billion people worldwide eat potato, and global crop production exceeds 300 MT per annum. Yet, this crop is particularly vulnerable to increased temperature, which is considered to be the most important uncontrollable factor affecting growth and yield (Levy and Veilleux, 2007). Potato (*Solanum tuberosum* L.) originated in the Andes of South America from regions with cool temperatures, and most cultivated germplasm is highly sensitive to elevated temperature. For most commercial cultivars, yield is optimal at average daytime temperatures in the 14–22 °C range, above which yield falls off sharply (Van Dam *et al*., 1996).

Elevated temperatures are known to affect numerous physiological processes in potato plants. Heat strongly suppresses tuberization and reduces the proportion of assimilated carbon partitioned to tuber starch (Ewing, 1981; Hancock *et al*., 2014; Wolf *et al*., 1991). Compounding this problem, photosynthetic performance is also adversely affected by high temperatures that reduce chlorophyll levels and CO_2_ fixation rates (Reynolds *et al*., 1990). In addition, high temperature has a negative effect on potato tuber dormancy causing premature sprouting or secondary growth (Bodlaender *et al*., 1964). A significant interaction is observed between photoperiod and temperature (Menzel, 1985). During longer day lengths, lower temperatures are required for optimal tuberization. However, effects of high temperature depend on the plant developmental stage; warm conditions can be beneficial during early growth phases whilst during tuber induction, cool temperatures especially during the dark period are essential (reviewed in Levy and Veilleux, 2007).

Whilst cultivated potato is generally a cool climate crop, there is significant variation in response to heat stress between cultivars (Levy, 1986; Levy *et al*., 1991; Marinus and Bodlaender, 1975; Mendoza and Estrada, 1979; Menzel, 1985; Midmore and Prange, 1991), in land races and wild potato species (Hetherington *et al*., 1983; Mendoza and Estrada, 1979; Reynolds and Ewing, 1989) and in progeny clones from heat‐tolerant parents (Haynes and Haynes, 1983; Mendoza and Estrada, 1979; Morpurgo *et al*., 1985; Veilleux *et al*., 1997). Despite reported variation, we are unaware of any reports that identify QTL for heat tolerance in potato. In contrast, in other crop species, QTL mapping studies have proven useful in identifying markers linked to heat stress tolerance. Multiple loci for heat tolerance have been identified in wheat (Paliwal *et al*., 2012) and maize (Messmer *et al*., 2009). A major QTL for high‐temperature germination and an additional QTL having smaller effects were identified in a genetic analysis of lettuce seed thermo‐inhibition (Argyris *et al*., 2008).

A major difficulty in screening for complex abiotic stress tolerance traits is control of environmental parameters whilst growing sufficient numbers of genotypes under replication for genetic analysis. In potato, segregation for earliness of tuberization is a confounding factor in heat stress screening and has led to the development of screens based on nodal cuttings (Ewing and Wareing, 1978; Van den Berg *et al*., 1990). This technique uses an excised potato leaf and its subtended axillary bud, that when maintained in moist compost, tuberizes rapidly. Leaf cuttings from induced plants produce tubers (in ~14 days) at the axillary bud, whereas non‐induced plants fail to tuberize. The nodal cutting assay therefore provides a convenient, space and time‐saving high‐throughput method to study tuberization and dry matter partitioning. The use of a model system for analysis of traits that are impacted on by multiple factors has the potential to simplify the trait and allows the investigator to focus on specific components.

In this study, we have exploited the well‐characterized 06H1 biparental diploid potato population (Prashar *et al*., 2014) to identify a locus associated with tuber yield at both conventional and mildly elevated temperature. Interestingly, this diploid population does not segregate for variation at the maturity locus on chromosome 5 (Kloosterman *et al*., 2013) showing fairly late uniform foliage maturity. A candidate gene encoding *HSc70* was identified within the QTL interval, and a particular allelic variant was linked to elevated yield in the 06H1 progeny. Expression of a transgene containing *HSc70* under its native promoter in the potato cultivar Desiree resulted in enhanced *HSc70* expression at elevated temperature leading to improved yield under moderately elevated temperature. Thus, our results identify *HSc70* expression level as a significant factor influencing yield stability under moderately elevated temperature and we identify specific allelic variants of *HSc70* for the induction of thermotolerance via conventional introgression or molecular breeding approaches.

## Results

### Assessing tuber yield using the nodal cutting assay and QTL analysis

**Figure 1:**
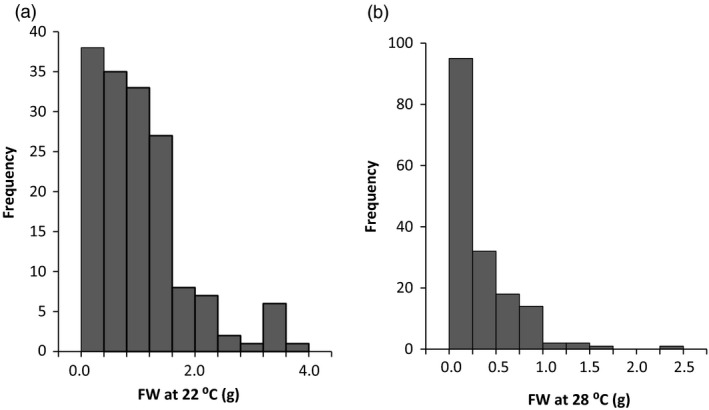
Frequency distribution of tuber yield from genotypes of the 06H1 population using the nodal cutting assay.(a) data from 22°C, (b) data from 28°C.

Nodal cutting tuberization assays were carried out for 170 genotypes from the 06H1 population. For each genotype, six nodal cuttings were prepared from two plants, using the second, third and fourth node from the apex. Two independent experiments were conducted. Nodal cutting tuber yields (based on a total of 12 cuttings) from the 06H1 population grown at 22 and 28 °C ranged from 0.0002 to 3.674 g (22 °C) and 0.0002–2.277 g (28 °C), with means of 1.017 and 0.285, respectively, the data being considerably skewed towards zero (Figure 1). These data were used for QTL analysis using the MapQTL 6 software (Van Ooijen, 2011) and the SNP linkage map previously reported (Prashar *et al*., 2014). This linkage map comprises 2157 mapped SNP markers generated using an Illumina SNP platform comprising a total of 8303 SNP markers. The 06H1 tuber yield data were subjected to a nonparametric Kruskal–Wallis (KW) analysis in MapQTL6 which essentially carries out a one‐way analysis of variance (ANOVA) to test for association between each mapped marker and the analysed trait. QTL effects were detected on three linkage groups, 1, 4 and 9. At 22 °C, the only QTL effect detected was around the maternal marker c2_11487 at 11.53 cM (K* = 14.21). At 28 °C, QTL were detected on linkage group 1 (biparental marker c2_49910 at 76.85 cM, K* = 21.32), on linkage group 4 (maternal marker c2_11487 at 11.53 cM, K* = 14.22) and on linkage group 9 (paternal marker c2_3948 at 11.64 cM, K* = 16.243).

### Identification of *HSc70* as a candidate gene for tuber yield in the tuber nodal cutting assay

The genes encoded in the region of the QTL on linkage groups 1, 4 and 9 were examined in the Potato Genome Browser hosted by Michigan State University (http://solanaceae.plantbiology.msu.edu/cgi-bin/gbrowse/potato/). For the linkage group 4, QTL inspection of the genomic region corresponding to ~5 cM either side of the QTL peak revealed a strong candidate gene encoding HSc70 (PGSC0003DMG400027750). Previously, genetic variation in *HSc70* has been suggested to underpin heat tolerance in cabbage (Park *et al*., 2013) and variation in heat‐shock protein levels have been correlated with heat tolerance in potato (Ahn *et al*., 2004). Inspection of a similar‐sized genomic region on linkage groups 1 and 9 revealed no obvious candidate genes. The presence of the *HSc70* gene in the QTL region on linkage group 4 allied to results from previous studies with this gene led to our targeting it for further functional analysis.

### Identification of *HSc70* alleles

**Figure 2:**
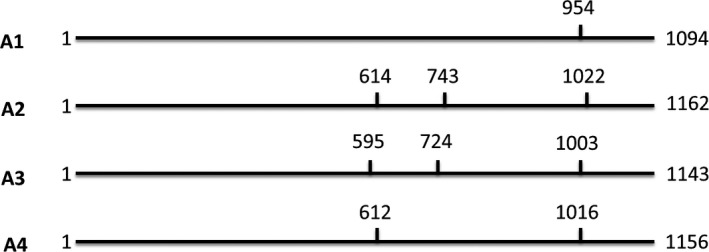
*HSc70* cleaved amplified polymorphic sequence (CAPS) assay. Four alleles were identified in the diploid parents. HB171(13) parent contains alleles A1 and A2 and 99FT1b5 contains alleles A3 and A4. Vertical lines represent HindIII restriction enzyme sites and numbers refer to nucleotide position.

**Figure 3:**
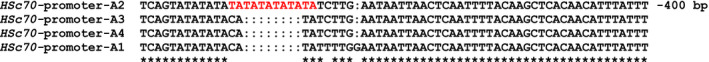
*HSc70* promoter sequence of the four different alleles. TA extension in A2 at 495 bp upstream of ATG start codon is marked in red. [Colour figure can be viewed at wileyonlinelibrary.com]

A region of the *HSc70* gene was amplified from the parents of the 06H1 population (HB171(13) and 99FT1b5) by PCR (Figure S1). Sequence analysis of the PCR products identified four distinct alleles with the HB171(13) parent containing alleles designated A1 and A2 and 99FT1b5 containing alleles A3 and A4. A cleaved amplified polymorphic sequence (CAPS) assay (Konieczny and Ausubel, 1993) was designed to discriminate between the four *HSc70* alleles (Figure 2), and the allelic complement of the genotypes from the 06H1 population was determined. For each genotypic class present in the population, the mean tuber fresh and dry weight yields from nodal cutting experiments were determined. Genotypes containing the A2 allele yielded the highest fresh weight at both 20 and 28 °C (*P* < 0.05), with the A2A3 genotype having the highest yields and the A1A4 combination the lowest yield (Table 1). The ‘phase’ of the marker data around the position of the gene on linkage group 4 is consistent with a maternally inherited QTL effect caused by action of the *HSc70* gene, the A2 allele being linked in coupling with the segregating maternal SNP alleles. The deduced amino acid sequences encoded by the four alleles were also compared (Figure S2). This analysis indicated that the amino acid sequences are similar and are only different in the C‐terminal substrate‐binding domain (SBD) (Mayer, 2013) (Figure S2). In the sequences encoded by the A2 and A3 alleles, there are six additional amino acids reside (KIEEVD) in this region compared with the proteins encoded by the A1 and A4 alleles. No amino acid sequence variation was specific to only one of the four alleles. To further dissect any functional significance of sequence variation, amplification of up to 1 KB upstream of the presumed ATG start codon was achieved for each allele by designing further PCR primers (Table S1). Comparison of the promoter sequences of the four alleles identified sequence differences specific to the A2 allele (Figure 3). In particular, at ca. 495 bp upstream of the ATG start codon, the A2 allele uniquely has an extended run of ten TA repeats compared with four repeats in the other three alleles (Figure 3).

**Table 1:** Genotype means after heat stress screening of 188 genotypes from the 06H1 population. Fresh weight (FW) and dry weight (DW) are measured in grams (g)

Trait measured	Genotypic mean for A1A3 (g)	Genotypic mean for A2A3 (g)	Genotypic mean for A1A4 (g)	Genotypic mean for A2A4 (g)
FW at 20 °C	2.27 ± 0.28^a^	3.58 ± 0.31^b^	1.46 ± 0.18^a^	3.13 ± 0.32^b^
FW at 28 °C	0.51 ± 0.13^ab^	1.24 ± 0.20^c^	0.35 ± 0.08^a^	0.92 ± 0.15^bc^
DW at 20 °C	0.50 ± 0.2^ab^	0.76 ± 0.18^b^	0.31 ± 0.22^a^	0.85 ± 0.18^b^
DW at 28 °C	0.11 ± 0.06^ab^	0.30 ± 0.08^c^	0.06 ± 0.01^a^	0.24 ± 0.08^bc^

Data are presented as mean ± standard error and different letters indicate significant differences between genotypes (Fisher's, *P* < 0.05).

### Expression levels of *HSc70* in genotypes with contrasting response to moderately elevated temperature

**Figure 4:**
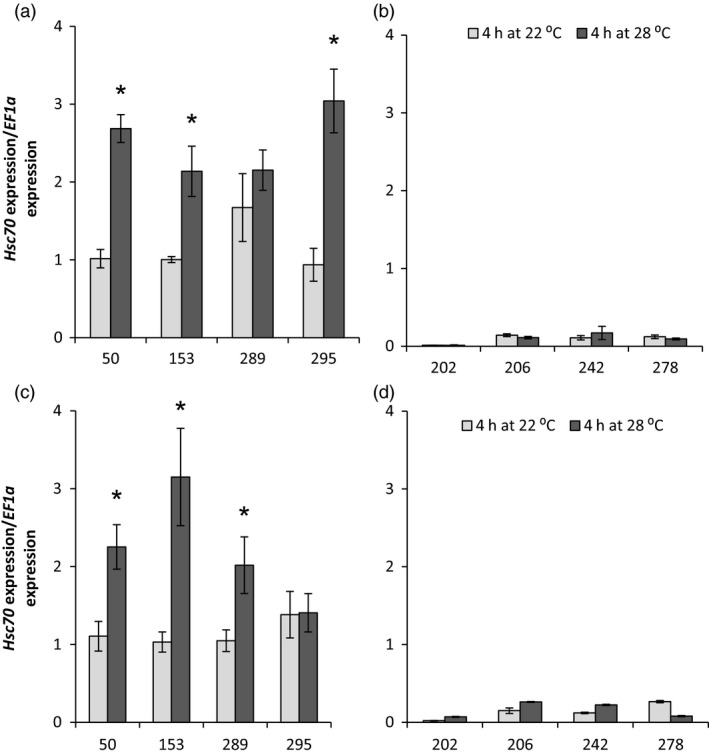
Relative expression level of *HSc70* in 06H1 population. (a) Tubers from A2A3 heat‐tolerant genotypes (genotype number 50, 153, 289 and 295); (b) tubers from A1A4 heat‐sensitive genotypes (genotype number 202, 206, 242 and 278); (c) leaves from A2A3 heat‐tolerant genotypes (genotype number 50, 153, 289 and 295) and; (d) leaves from A1A4 heat sensitive genotypes (genotype number 202, 206, 242 and 278). Asterisk indicates statistical differences between temperatures as determined by Student's *t*‐test. Error bars represent the standard error of the mean (*n* = 3) (*P* < 0.05).

*HSc70* expression level was compared by qRT‐PCR in four genotypes that contained the A2A3 allelic combination and gave good tuber yield in the nodal cutting assay at 28 °C and with four low‐yielding genotypes containing A1A4 alleles (Figure 4). In both tubers and leaves, *HSc70* expression was significantly higher in the A2A3 genotypes than in the A1A4 when maintained at the optimal temperature of 22 °C (*P* < 0.05 for both organs). Furthermore, following transfer of cuttings to 28 °C for 4 h, genotypes containing the A2A3 alleles exhibited a further significant increase in the abundance of the *HSc70* transcript in both leaves and tubers, whereas there was no significant change in abundance (*P* = 0.8 to 0.3) of the same transcript in genotypes containing the A1A4 alleles (Figure 4).

In order to determine which *HSc70* allele was up‐regulated on exposure to elevated temperature, a semi‐quantitative RT‐PCR expression assay was carried out and gave similar results to those shown in Figure 4 (Figure S3a). The PCR products from the RT‐PCR assay were sequenced, and from eight cloned products, sequence analysis showed that all were transcripts arising from the A2 allele (Figure S3b).

### Transient expression of *HSc70* in *Nicotiana benthamiana* leaves

**Figure 5:**
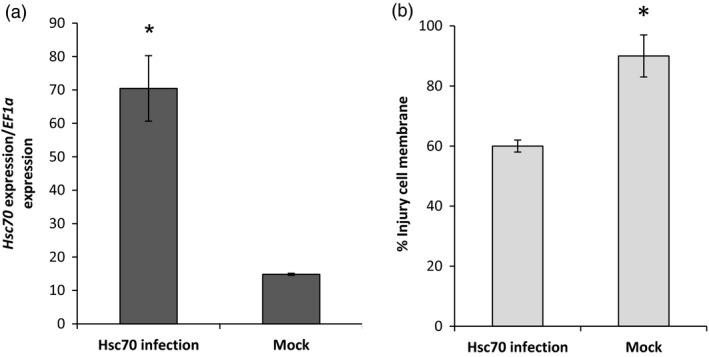
Agro‐infiltration of *Nicotiana benthamiana* plants. (a) Relative expression level of *HSc70* after 1 day at 45 °C in agro‐infiltrated plants with *HSc70* construct versus agro‐infiltrated plants with empty vector (Mock). (b) Membrane damage in agro‐infiltrated *N. benthamiana* plants after 1 day at 45 °C by electrolyte leakage assay compared with agro‐infiltrated plants with empty vector (Mock). Asterisks indicate significant difference with wild type at high temperature as estimated using Student's *t*‐test (*P* < 0.05). Error bars represent the standard error of the mean (*n* = 6) (*P* < 0.05).

The function of the A2 *HSc70* allele was characterized in transient expression experiments conducted in *N. benthamiana*. A binary construct containing the A2 coding sequence and 1 KB of sequence upstream of the ATG start codon was engineered and introduced into leaves of *N. benthamiana* by agro‐infiltration. Following agro‐infiltration, plants were placed in a chamber at 45 °C under 12 h light. After 24 h, the *HSc70* expression level was compared in agro‐infiltrated plants and mock‐inoculated plants. Those agro‐infiltrated with the *HSc70* construct expressed the *HSc70* gene at levels ca. sevenfold higher than controls. Furthermore, plants agro‐infiltrated with the *HSc70* construct exhibited significantly lower levels of cell membrane injury compared with controls (60% compared with 90%, *P* < 0.05, Figure 5). The values were consistent with the visual assessment of plant damage which was greater in controls (Figure S4).

### Promoter deletion analysis of the A2 *HSc70* allele

**Figure 6:**
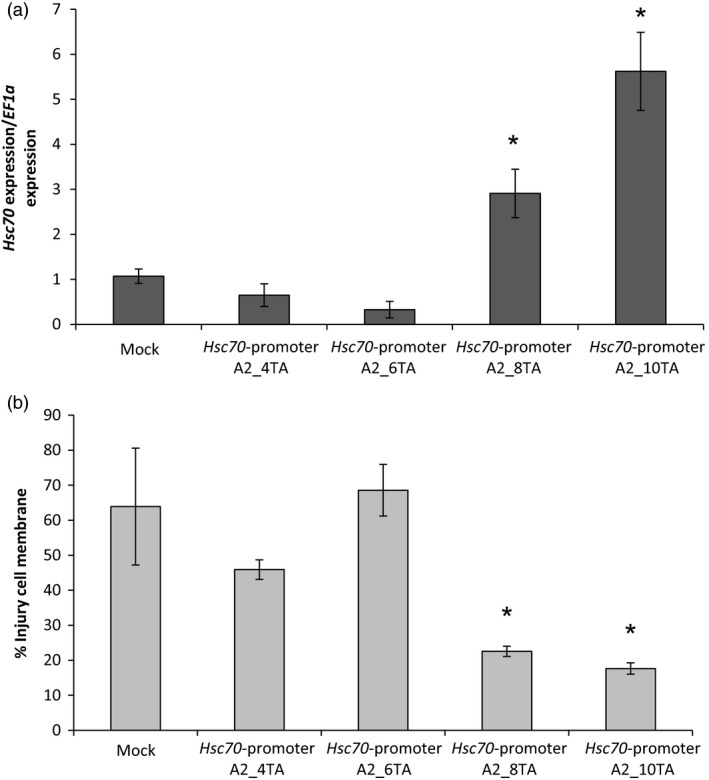
Promoter deletion analysis by agro‐infiltration of *Nicotiana benthamiana* plants. (a) Relative expression level of *HSc70* after 1 day at 42 °C in agro‐infiltrated plants with *HSc70* promoter deletion constructs versus agro‐infiltrated plants with empty vector (Mock). (b) Membrane damage in agro‐infiltrated *N. benthamiana* plants after 1 day at 42 °C by electrolyte leakage assay compared with agro‐infiltrated plants with empty vector (Mock). Asterisks indicate significant difference. Error bars represent the standard error of the mean (*n* = 3) (*P* < 0.05).

The A2 allele was differentiated from the other three *HSc70* alleles by the presence of ten TA repeats in the promoter in a region 495 nucleotides upstream of the deduced start codon, whereas in the A1, A3 and A4 alleles only four TA repeats were present in this region (Figure 3). In order to test whether this repeat sequence was connected to the elevated *HSc70* expression level observed for the A2 allele on exposure to elevated temperature, promoter deletions were tested. Promoter constructs with 4 to 10 TA repeats were engineered in a binary construct upstream of the A2 coding sequence. Transient expression of *HSc70* was measured on agro‐infiltration of these constructs into *N. benthamiana* leaves following exposure to elevated temperature. *HSc70* expression level was significantly higher (sixfold) in plants agro‐infiltrated with constructs containing eight or 10 TA repeats than in those agro‐infiltrated with constructs containing four or six repeats (Figure 6a). Furthermore, the cell membrane injury level was greater in the plants agro‐infiltrated with constructs containing four or six repeats (Figure 6b), providing evidence that the TA repeat sequence is important for *HSc70* expression and reinforcing the link between *HSc70* expression and heat tolerance. The values were consistent with the visual assessment (Figure S7).

### Characterization of transgenic potato lines overexpressing the A2 *HSc70* allele

**Figure 7:**
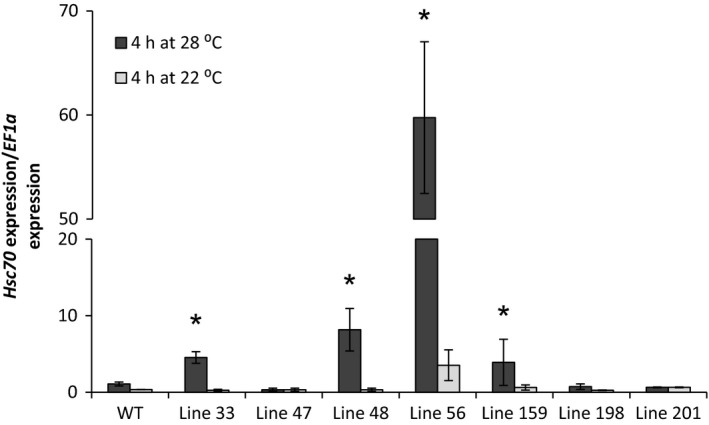
Expression level of *HSc70* gene in transgenic potato lines A2 *HSc70* allele after 4 h at 22 °C or 28 °C relative to wild‐type plants Desiree cv (WT). Asterisks indicate significant difference. Error bars represent the standard error of the mean (*n* = 3) (*P* < 0.05).

Transgenic potato lines expressing the A2 *HSc70* allele under its native promoter were generated in the cultivar Desiree and independent transgenic lines were screened for *HSc70* expression level in leaves (Figure 7). In leaves harvested from plants grown at 22 °C, *HSc70* expression level was low in both transgenic lines and controls although one transgenic line (line 56) did exhibit significantly higher *HSc70* expression at this temperature. In plants subjected to 4 h of elevated temperature (28 °C), there was a dramatic increase in *HSc70* expression level particularly in line 56, where *HSc70* transcripts were 60‐fold (*P* < 0.05) more abundant than in wild‐type controls. Lines 33 and 48 exhibited significantly greater expression levels (four‐ and eightfold, respectively) than wild type after elevated temperature treatment. Lines 33, 48 and 56 were therefore selected for further analysis.

**Figure 8:**
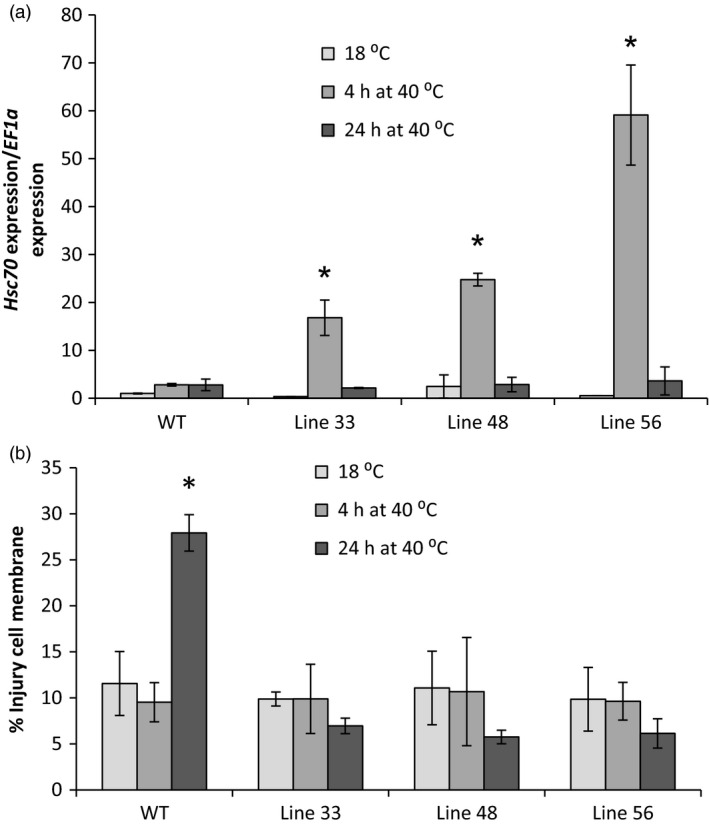
Screening of overexpression transgenic lines at high temperature (40 °C). (a) Expression level of *HSc70* gene in transgenic potato lines A2 *HSc70* allele and wild‐type plants Desiree cv (WT). (b) Membrane damage by electrolyte leakage assay of A2 HSc70‐expressing lines and wild‐type plants Desiree cv (WT). Asterisk indicates statistical differences with wild type at high temperature as determined by Student's *t*‐test. Error bars represent the standard error of the mean (*n* = 6).

Wild‐type and selected A2 *HSc70‐*expressing lines were exposed to elevated temperature (40 °C) for 24 h. Leaf cell membrane injury was assessed using an electrolyte leakage assay. After 4 h, *HSc70* expression was greatly enhanced by up to 50‐fold in all transgenic lines relative to wild type. However, following 24 h exposure, there was no significant difference in expression level between any of the lines tested, with expression level in the transgenic lines declining to the levels prior to heat exposure (Figure 8a). No changes in electrolyte leakage from leaves were observed after 4‐h high‐temperature exposure in any of the genotypes; however, after 24 h, there was significant increase in electrolyte leakage from wild‐type leaves that was not observed in any of the *HSc70* transgenic lines (Figure 8b).

### Protection of tuber yield at elevated temperature in A2 *HSc70*‐expressing lines

**Figure 9:**
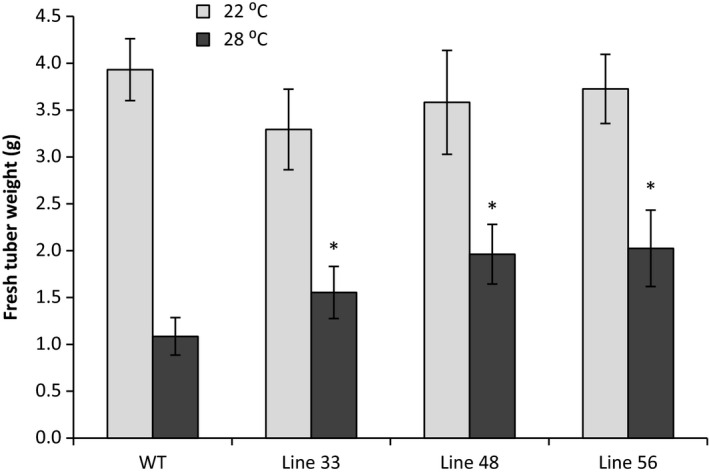
Fresh weight of tubers in A2 *HSc70*‐expressing lines at 22 and 28 °C. Asterisk indicates statistical difference with wild type at high temperature (Fisher's, *P* < 0.05). Error bars represent the standard error of the mean (*n* = 3) (*P* < 0.05).

Having established that elevated transient expression of A2 *HSc70* provided a temperature‐dependent protective effect as assessed by the electrolyte leakage assay, we wished to determine whether expression of the A2 allele could also protect tuber formation and yield at moderately elevated temperature. We therefore performed nodal cutting tuber yield assays. At 20 °C, yield was not significantly (*P* < 0.05) different between the wild‐type and any of the three transgenic lines tested (Figure 8). However, in Desiree WT, yield decreased by 75% at 28 °C compared to 22 °C (*P* < 0.05). Yield reductions were also observed in transgenic lines overexpressing *HSc70* at higher temperatures; however, these were not as extensive as observed in Desiree wild type (Figure 9). For example in lines 48 and 56, fresh weight yield at 28 °C was ca. twofold greater than in wild‐type controls at the same temperature (*P* < 0.05). Dry weight yield values also showed the same significant pattern with higher yield in the transgenic lines at elevated temperature (Table S3). Tuber yield was also measured in plants grown from stem cuttings. At maximum day temperature of 20 °C, two of the three transgenic lines exhibited no significant difference in tuber yield between the overexpressing lines and wild‐type control whilst line 48 exhibited a significant increase in both fresh and dry tuber weight. In contrast, at 28 °C, tuber yield was significantly higher (*P* < 0.05) in all of the transgenic lines relative to the controls on both a fresh weight and dry weight basis (Table 2).

**Table 2:** Weight of tubers from single‐stem plants of A2 *HSc70*‐expressing lines. Fresh weight (FW) and dry weight (DW) are measured in grams (g)

Trait measured	Line 33 (g)	Line 48 (g)	Line 56 (g)	WT (g)
FW at 20°C	11.9 ± 4.0^a^	14.7 ± 4.9^b^	11.2 ± 3.8^a^	10.6 ± 2.1^a^
FW at 28°C	15.3 ± 5.1^b^	19.7 ± 8.8^b^	17.6 ± 4.9^b^	8.8 ± 1.8^a^
DW at 20°C	1.89 ± 0.63^a^	2.93 ± 0.98^b^	1.7 ± 0.57^a^	1.96 ± 0.30^a^
DW at 28°C	2.7 ± 0.90^b^	4.80 ± 1.00^c^	2.90 ± 0.73^b^	1.39 ± 0.27^a^

Data are presented as mean ± standard error (*n* = 9) and different letters indicate significant differences between lines (Fisher's, *P* < 0.05).

## Discussion

### Genetic analysis of tuber yield using nodal cuttings

For many years, a simplified model of the potato plant based on nodal cuttings has been used as a convenient system for studying tuberization (Kumar and Wareing, 1972, 1974). It has also been suggested that cuttings can be used as a screen for heat tolerance (Ewing and Wareing, 1978). In addition, nodal cutting assays were developed to investigate the effects of temperature on tuber second growth (Van den Berg *et al*., 1990) where the negative effects of temperature on tuberization were clearly demonstrated. Heat tolerance is dependent on both the ability to produce haulm that grows vigorously at high temperature and on maintaining carbon partitioning and tuber development. For genetic analysis, it is useful to dissect complex traits into their components. In view of recent advances in potato genetics such as the availability of biparental populations and dense genetic maps (Prashar *et al*., 2014), we considered it timely to investigate the genetic control of tuber yield by deploying a nodal cutting system on a segregating population.

### *HSc70* identification from genetic analysis

In the diploid 06H1 population, QTL for tuber yield were identified on linkage group 4 and two other linkage groups. The linkage group 4 QTL for yield were detected at both temperatures tested (22 and 28 °C). Inspection of the genome browser indicated that there were a large number of genes in the linkage group 4 QTL region and so the approach did not yield definitive genetic evidence of the causative gene. Nevertheless, it did focus our attention on the set of candidate genes amongst which a gene encoding HSc70 was considered a good candidate for yield protection particularly at the elevated temperature.

Heat‐shock cognate protein 70 (HSc70) is a constitutively expressed molecular chaperone which belongs to the heat‐shock protein 70 (HSP70) family (Al‐Whaibi, 2011). In contrast to heat‐shock proteins (HSPs), heat‐shock cognate proteins are not generally induced by elevated temperature (McCallister *et al*., 2015). Despite this difference in expression response, no amino acid motifs have been defined that distinguish heat‐shock cognate proteins from HSPs. Phylogenetic analysis confirms that all four alleles of the presumed *HSc70* gene in this study are more closely related to other *HSc70* genes in Arabidopsis and tomato than to *HSP70* sequences (Figure S5). Nevertheless, the A2 allele identified here is clearly heat inducible, illustrating the difficulties in annotating this gene family. All organisms respond to heat stress by inducing heat‐shock proteins (HSPs). HSP70s are the most abundant type of HSP, having important roles in preventing newly synthesized proteins from misfolding and aggregating. HSP70s are encoded by multigene families that can be divided into four subfamilies based on subcellular localization: cytosol, endoplasmic reticulum (ER), plastids and mitochondria (Sung and Guy, 2003). In addition, *Nicotiana tabacum* contains a nuclear‐localized HSP70, NtHSP70‐1, which helps to prevent the fragmentation and degradation of nuclear DNA during heat stress (Cho and Choi, 2009). Although many studies have elucidated the molecular functions of individual family members, genomewide analysis of this family is still limited, especially for crop species (Jung *et al*., 2013).

### *HSc70* and protection from elevated temperature

HSc70s are characterized by highly conserved ATPase and substrate‐binding domains (SBDs) (Mayer, 2013). ATP binding by the ATPase domain induces conformational changes in the SBD, facilitating transient association with hydrophobic stretches in peptides. When stimulated by both substrate‐binding and the J‐domains of co‐chaperone proteins, HSc70s hydrolyse ATP to ADP, triggering a SBD conformation change resulting in capture of the hydrophobic substrate. Release of ADP by nucleotide exchange factors (NEFs) causes the SBD to return to the open conformation, releasing the substrate.

In Arabidopsis, overexpression of *HSc70* correlates with the acquisition of thermotolerance as well as an increase in tolerance to water deficit and salt stress (Alvim *et al*., 2001; Leborgne‐Castel *et al*., 1999; Lee and Schöffl, 1996; Sung and Guy, 2003). Attempts to decrease *HSc70* expression levels using a transgenic approach were unsuccessful implying that reduced *HSc70* expression is lethal. Constitutive overexpression resulted in transgenic lines with impaired developmental phenotypes characterized by dwarfism and altered root structure. Nevertheless, these transgenic lines also exhibited a greater tolerance to heat shock (44 °C for 10 min) than controls (Sung and Guy, 2003). These results led to the conclusion that it is necessary to tightly control *HSc70* expression during development to avoid pleiotropic effects whilst having good tolerance to abiotic stresses. In the present study, we examined allelic variation both in the coding region and in the upstream promoter sequence of *HSc70*. Comparison of the deduced amino acid sequence encoded by the four alleles revealed differences in the coding region (Figure S2) although none specific to the A2 encoded protein. The A2 and A3 alleles contain the C‐terminal sequences KIEEVD which are missing in the A1 and A4 alleles. The conserved C‐terminal EEVD sequences of Hsp70 and Hsp90 mediate interactions with specialized tetratricopeptide repeat (TPR) domains in Hop and other related co‐chaperones (Brychzy *et al*., 2003). The absence of this motif in the A1 and A4 alleles could be of functional significance and could account for the particularly low level of heat tolerance (based on yield at elevated temperature, Table 1) in the A1A4 genotype class. However, the A1A3 genotype class, containing one allele with the C‐terminal domain also exhibits lower yields than genotypes containing the A2 allele.

Interestingly, the protective A2 allele exhibited a unique extended TA repeat approximately 495 bp upstream of the putative ATG start codon, implying that the A2 phenotype may be due to differences in expression patterns at elevated temperature. The best combination of alleles for enhanced yield arises from the A2A3 combination, where both alleles contain the KIEEVD domain as well as the high level of expression from the A2 allele and so it is possible that a combination of the A2 promoter sequence and the presence of the KIEEVD domain in both alleles are required for optimal yield.

In Desiree lines transformed with the construct in which expression was driven by the A2 promoter, *HSc70* expression was similar in wild‐type and transgenic lines grown at 22 °C; however, on transfer to elevated temperature, A2 expression was rapidly and transiently induced in transgenic lines. Transgenic lines did not exhibit any developmental abnormalities and our work therefore supports previous observations that appropriate *HSc70* expression is essential to provide protection against abiotic stress whilst preventing adverse pleiotropic phenotypes. Our work further highlights the A2 promoter as a tool to drive heat‐inducible expression of a range of gene constructs in potato and other Solanaceae.

We are unaware of any previous reports that identify alleles of *HSc70* genes that may confer different expression properties and hence impact on yields particularly under abiotic stress conditions. However, in this respect, reports of heat tolerance in cabbage genotypes are of relevance (Park *et al*., 2013). Heat‐responsive gene expression profiles in four heat‐tolerant and four heat‐sensitive genotypes were compared. Significantly higher expression levels (ca. eightfold) of an *HSc70* gene were measured in all heat‐tolerant lines than in the heat‐sensitive lines, implying that *HSc70* may be a marker for heat tolerance. Differential expression of small HSPs has been reported for several plant species including potato, where genotypic differences in heat tolerance correlated with small HSP expression. Two thermotolerant potato varieties expressed higher levels of small HSPs than two more sensitive varieties (Ahn *et al*., 2004). Similarly, in common bean, heat‐tolerant varieties express small HSPs at higher levels on exposure to heat stress (Simões‐Araújo *et al*., 2003).

### Promoter motif associated with the differential response of the A2 allele

To account for the different expression characteristics of the A2 allele of *HSc70*, the promoter sequences of the four different alleles in the parents of the 06H1 population were compared. The most striking difference between the promoter sequences was an extended TA repeat motif in the A2 promoter which contained 10 repeats compared with four in the other alleles. In yeast, it has been demonstrated that tandem TA repeats in promoter regions can result in enhanced expression levels (Vinces *et al*., 2009) due to effects on local chromatin structure. Whilst we are unaware of a specific effect of TA tandem repeats in plants in relation to heat stress, TA‐rich regions in plant promoters do often enhance gene expression (Sandhu *et al*., 1998). In this study, promoter deletions clearly demonstrate the relationship between the number of TA repeats and the *HSc70* expression level on exposure to elevated temperature in transient assays in *N. benthamiana*. It will be interesting to investigate the occurrence of the TA repeat allele in other potato genotypes and wild species.

## Conclusion

Overall, our results demonstrate that the presence of the A2 *HSc70* allele confers tolerance to elevated temperature, both in the 06H1 population and transgenic lines where *HSc70* expression is driven by the A2 promoter. In the nodal cutting system and in plants generated from stem cuttings, the presence of the A2 allele results in enhanced yield at elevated temperature. In transgenic lines, the yield was up to twofold greater than in wild‐type plants at 28 °C but with no significant effect on yield at 20 °C. In 06H1 genotypes, however, yield was enhanced at both temperatures tested. In both cases, we propose the rapid response of the A2 allele to temperature perturbation underlies the yield effect. Deployment of the A2 allele in potato breeding may provide a strategy for enhancing yield either under temperate conditions or under periods of abiotic stress.

## Experimental procedures

### 06H1 population

The mapping population used here (06H1) is a full‐sibling progeny (*n* = 186) of a cross between two highly heterozygous diploid potato clones (HB171(13) and 99FT1b5), both of which result from crosses between diploids of *Solanum tuberosum* group Tuberosum and *S. tuberosum* group Phureja (Prashar *et al*., 2014).

### Growth of plant material

06H1 clones were grown from cores (6 mm diameter, excised from tubers, each containing a single bud) in 10‐cm‐diameter pots containing standard compost mix. Plants were raised in a glasshouse maintained at a daytime temperature of 20 °C and a nocturnal temperature of 15 °C. Light intensity (photosynthetic photon flux density) ranged from 400 to 1000 μmol m^−2^ s^−1^. Single nodal cuttings (Ewing and Wareing, 1978) were taken from 7‐ to 8‐week‐old plants and the base of the petiole was placed in 50/50 coir/sand mix. A cutting consists of a fully extended leaf and its subtended bud. Cuttings were left in glasshouse conditions for 24 h then moved to growth rooms set at 70% humidity, 12‐h photoperiod (light intensity of 400 μmol m^−2^ s^−1^) and various temperature regimes. Cuttings were watered daily with prewarmed water. Tubers were harvested after 3 weeks.

Four heat‐sensitive and four heat‐tolerant genotypes of the 06H1 population were grown from cores excised from tubers as described above. Plants were raised under glasshouse conditions for 8 weeks. Then, the plants were acclimated for 2 weeks under controlled environment conditions (20 °C day/16 °C night). Plants were moved to a cabinet at high temperature (28 °C) and low temperature (22 °C) for 4 h, at a light intensity of 400 μmol m^−2^ s^−1^. After 4 h, leaves and tubers were collected, immediately frozen in liquid nitrogen, and then stored at −80 °C until use.

*HSc70*‐overexpressing lines were grown from in vitro‐propagated tissue culture plants in 10‐cm‐diameter pots containing standard compost mix. Plants were grown in a glasshouse (15–20 °C, 12 h light, 400 to 1000 μmol m^−2^ s^−1^) for 7–8 weeks. Single nodal cuttings were prepared as described above.

### QTL analysis

QTL mapping of nodal cutting tuber yield data (22 and 28 °C) was performed using MapQTL^®^6.0 (Van Ooijen, 2011) and Genstat 15.1 (VSN International Ltd.) software. The nonparametric Kruskal–Wallis (KW) test supported in MapQTL version 6.0 was performed. In this method, a single marker analysis was used to test the association of a marker with the trait at significance *P* ≤ 0.001.

### Allele mining and cleaved amplified polymorphic sequence (CAPS) assay

Genomic DNA was isolated from leaves using the AquaGenomic™ DNA isolation solution (http://www.aquaplasmid.com) according to manufacturer's instructions. PCR primers (Table S1) were designed to amplify a partial fragment from parental DNA templates. PCR was performed on 25–50 ng DNA in 50 μL reaction volume using 2.5 U of Platinum high‐fidelity DNA polymerase and buffer (www.invitrogen.com) containing 1.25 mm MgSO_4_. Gene‐specific primers and deoxynucleotides (dNTPs) were used at a concentration of 0.4 and 200 μm, respectively. Thermal cycling conditions were as follows: 2‐min denaturation at 95 °C followed by 25–40 cycles (30 s at 95 °C, 30 s annealing at the appropriate Tm, 1 to 2‐min extension at 68 °C) followed by 5‐min final extension step at 68 °C. Polymorphisms were present in the parental sequences at positions that resulted in the presence or absence of a *Hin*dIII restriction site (Figure 2). PCR products were digested with *Hin*dIII prior to being analysed by electrophoresis on agarose gels and visualized following staining with ethidium bromide. Distinct restriction digestion patterns were observed for the four alleles identified (Figure 2).

### qRT‐PCR

RNA was extracted from potato leaves and tubers as described (Ducreux *et al*., 2008). The first‐strand cDNA templates were generated by reverse transcription, using random hexamers as primer and SuperScript II reverse transcriptase (Invitrogen Life Technologies, Carlsbad, CA). Potato elongation factor1‐alpha (*EF1α*) primers were used as a control. The expression level of *HSc70* was analysed using the StepOnePlus Real‐Time PCR system (Applied Biosystems) and StepOne Software version 2.3 (Applied Biosystems, Foster City, CA, USA). Gene‐specific primers and Universal probe Library (UPL, Roche Life Science) probes (Table S2) were used at a concentration of 0.2 and 0.1 μm, respectively. Thermal cycling conditions were as follows: 10‐min denaturation at 95 °C followed by 40 cycles of 15 s at 94 °C, 60 s at 60 °C. Relative expression levels were calculated and the primers validated using the Ct method (Livak, 1997). To normalize the values, an alternative method for calculating relative quantification was used (Pfaffl, 2001). The *HSc70* expression level in transgenic lines was determined using the same method.

### *HSc70* Promoter analysis

Genomic DNA was isolated and PCR performed as described for allele mining using the specific primers described in Table S1. PCR products were analysed by electrophoresis on agarose gels and DNA fragments eluted from the gel. The purified DNA fragments were ligated into pGEM‐T and transformed into *Escherichia coli* strain DH5α, and the plasmid DNA from each clone was extracted using a DNA Plasmid Miniprep Kit (Promega, Madison, WI) and sequenced with M13 universal primers on a 3730 automated DNA sequencer (Applied Biosystems: http://www.lifetechnologies.com) using a cycle sequencing protocol and the BigDye Terminator Cycle Sequencing Kit (version 3.1; Applied Biosystems). Analysis of sequences was performed using Sequencher software v.4.9 (http://genecodes.com/).

### Transgenic plants generation

The binary construct was designed using the GoldenBraid strategy (gb.cloning.org) (Sarrion‐Perdigones *et al*., 2011). Specific primers for the *HSc70* promoter from the A2 allele and the *HSc70*‐A2 coding region containing the required BsmBI type II restriction sites were designed (Table 1). PCR amplification was performed using High‐Fidelity Polymerase (Invitrogen). PCR products were analysed by agarose 1% gel electrophoresis and purified using a Promega kit. The PCR‐purified fragments were ligated into a pUPD entry vector using the BsmBI digestion–ligation reaction protocol of the GoldenBraid 2.0 cloning methodology. This method required repeated cycles of 37 °C for 2 min and 16 °C for 5 min. Once complete, the ligation reaction was transformed into *E. coli* strain DH5α (Invitrogen). Cloned inserts were sequenced using M13 universal primers to verify the fragment sequence and the type II restriction sites required for recombination in the expression vector. The *HSc70* overexpression construct in pDGB3 was created by recombining GBparts (*HSc70*‐promoter, *HSc70*‐coding region, pTnos‐terminator) as described by Sarrion‐Perdigones *et al*. (2011) using BsaI (New England Biolabs, Ipswich, MA) restriction enzyme and T4 DNA ligase in 25‐cycle digestion/ligation reactions. The binary construct was transformed into *Agrobacterium tumefaciens* strain AGL1 by electroporation (Curtis, 2004) Transformation of potato cv. Desiree using this construct was as described (Ducreux *et al*., 2005).

### Electrolyte leakage assay

Cell membrane injury was assessed using an electrolyte leakage assay as described in Campos *et al*. (2003). Four replicate samples of three 10‐mm leaf discs were punched from each leaf sample assayed and placed in a test tube. The discs were washed twice with deionised water, and then, 5 mL of deionised water was added to each tube and samples were shaken for 1 h in a 29 °C incubator. The leachate was transferred to a 50‐mL tube, 25 mL deionised water was added and the initial conductivity was measured using a conductivity meter. Samples were autoclaved, and total conductivity determined after cooling to room temperature. The extent of cell membrane injury was calculated as follows: [Initial conductivity × 100/total conductivity].

### Transient expression in *N. benthamiana*

For the transient expression experiments, plasmids were transferred to *A. tumefaciens* strain AGL1 by electroporation. Agro‐infiltration was performed as described previously (Wydro et al., 2006). Overnight‐grown bacterial cultures were pelleted and re‐suspended in agro‐infiltration medium (10 mm MES, pH 5.6, 10 mm MgCl_2_, and 200 mm acetosyringone) to an optical density at 600 nm of 1.2. Infiltrations were carried out using a needle‐free syringe in leaves of *N. benthamiana* plants (growing conditions: 24 °C day/20 °C night in a 16 h‐light/8 h‐dark cycle), and the plants were kept at 22 °C overnight. The following morning, the agro‐infiltrated plants were moved to 45 °C. After 24 h, 12‐h photoperiod with light intensity of 400 μmol m^−2^ s^−1^, leaves were harvested for gene expression analysis and cell membrane injury assays as described above.

### Promoter deletion assays

To investigate the role of the TA repeats in the A2 promoter in the region 495 base pairs upstream of the start codon, promoter deletions were engineered and the effects tested on transient expression in *N. benthamiana*. Sequences from the *HSc70* promoter containing four, six, eight or 10 TA repeats were engineered using GenArt™ Gene Synthesis (Invitrogen) as illustrated in Figure S6. The binary constructs were generated using the GoldenBraid strategy as described previously, and plasmids were transferred to *A. tumefaciens* strain AGL1 by electroporation. The transient expression experiments in *N. bethamiana* were performed as described previously, and leaves were harvested for gene expression analysis and cell membrane injury assays as described above.

### Statistical analysis

All analysis of variance (ANOVA) and the Student's *t*‐test were conducted using GenStat, 18th edition (VSN International, Oxford, UK).

## Conflict of interest

The authors declare no conflict of interest.

## Supporting information


**Figure S1** Clustal Omega alignment of the genomic DNA sequences of HSc70 alleles A1, A2, A3 and A4 isolated from parents of the 06H1 population (HB171(13) and 99FT1b5). Intron is indicated by blue text. Quantitative PCR primers and probe binding sites are highlighted in grey. Reverse primers used to amplify promoter regions: HSC70PA1R CTGAACGAGAATCATGAATCT; HSC70PCOMMONR AGATGCGAAGCGATTAATTGGT; HSC70PA3R TATACCAAACATAAACTCAT; HSC70PA4R TCCTAGCTCCAATACTAAACA.
**Figure S2** CLUSTAL O (1.2.1) multiple sequence alignment of the predicted *HSc70* amino acid sequences from the C‐terminal region of four alleles.
**Figure S3** Expression level of *HSc70* by semi‐quantitative RT‐PCR. (a) Bands of semi‐quantitative RT‐PCR in agarose gel (1%). HT means heat tolerante genotype; HS means heat sensitive genotype; C means control, L means 4 h at 20 °C and H measn 4 h at 28 °C. (b) Sequence analysis of clone products with *HSc70*‐A2 sequence and *HSc70*‐A3 sequence as model, where all transcripts show GATT region as A2.
**Figure S4** Phenotype of *HSc70* agro‐infiltrated and Mock inoculated plants of *Nicotiana benthamiana* after 24 h at 45 °C.
**Figure S5** Phylogenetic tree of heat shock protein sequences resulting from a BLASTP search against Arabidopsis, Potato and Tomato databases using the translated *HSc70* A2 allele. The tree was generated using Phylogeny.fr web service (Dereeper *et al*., 2008). The scale bar represents amino acid substitutions per site, that is the number of changes or ‘substitutions’ divided by the length of the sequence.
**Table S1** Primer sequences used for PCR and binary construct.
**Table S2** Primer sequences and probe used in qRT‐PCR.
**Table S3** Nodal cutting results of *HSc70* overexpression lines. Fresh weight (FW), dry weight (DW).
