# Supplementary material for: Engineering heat tolerance in potato by temperature‐dependent expression of a specific allele of HEAT‐SHOCK COGNATE 70
Source: Plant Biotechnol J. 2017 Jun 20;16(1):197–207. doi: 10.1111/pbi.12760 (PMC5785350; doi:10.1111/pbi.12760)
Supplement: Supplementary file 1 — Figure S1 Clustal Omega alignment of the genomic DNA sequences of HSc70 alleles A1, A2, A3 and A4 isolated from parents of the 06H1 population (HB171(13) and 99FT1b5). Intron is indicated by blue text. Quantitative PCR primers and probe binding sites are highlighted in grey. Reverse primers used to amplify promoter regions: HSC70PA1R CTGAACGAGAATCATGAATCT; HSC70PCOMMONR AGATGCGAAGCGATTAATTGGT; HSC70PA3R TATACCAAACATAAACTCAT; HSC70PA4R TCCTAGCTCCAATACTAAACA. Figure S2 CLUSTAL O (1.2.1) multiple sequence alignment of the predicted HSc70 amino acid sequences from the C‐terminal region of four alleles. Figure S3 Expression level of HSc70 by semi‐quantitative RT‐PCR. (a) Bands of semi‐quantitative RT‐PCR in agarose gel (1%). HT means heat tolerante genotype; HS means heat sensitive genotype; C means control, L means 4 h at 20 °C and H measn 4 h at 28 °C. (b) Sequence analysis of clone products with HSc70‐A2 sequence and HSc70‐A3 sequence as model, where all transcripts show GATT region as A2. Figure S4 Phenotype of HSc70 agro‐infiltrated and Mock inoculated plants of Nicotiana benthamiana after 24 h at 45 °C. Figure S5 Phylogenetic tree of heat shock protein sequences resulting from a BLASTP search against Arabidopsis, Potato and Tomato databases using the translated HSc70 A2 allele. The tree was generated using Phylogeny.fr web service (Dereeper et al., 2008). The scale bar represents amino acid substitutions per site, that is the number of changes or ‘substitutions’ divided by the length of the sequence. Table S1 Primer sequences used for PCR and binary construct. Table S2 Primer sequences and probe used in qRT‐PCR. Table S3 Nodal cutting results of HSc70 overexpression lines. Fresh weight (FW), dry weight (DW). [file PBI-16-197-s001.docx]

**Table S1**. Primer sequences used for PCR and binary construct

| Name | Forward | Reverse |
| --- | --- | --- |
| *Hsc70a* | CCAAAAACAATCTGTAATTCTGTGA | AGCCTCAGCAATCTCCTTCA |
| *HSc70*p750 | GTATAAACCAACATCTTGG |  |
| *HSc70*_A1 |  | CTGAACGAGAATCATGAATCT |
| *HSc70*_A3 |  | TATACCAAACATAAACTCAT |
| *HSc70*_A4 |  | TCCTAGCTCCAATACTAAACA |
| *HSc70*_Common |  | ACCAATTAATCGCTTCGCATCT |
| *HSc70*p1000 | gccccaattcttactttttgg | ccttcaccttttccggccat |
| *HSc70*cd | CCAAAAACAATCTGTAATTCTGTGA | TGAAGGAGATTGCTGAGGCT |
| *HSc70*p1000_GB | GCGCCGTCTCGCTCGAATGGCC  GAAAAGGTGAAGG | GCGCCGTCTCGCTCGAAGCT  TAATCAACTTCTTCAATCTTAGGG |
| *HSc70*cd_GB | GCGCCGTCTCGCTCGGGAGGCG  TACCTCTTACTTTTTGG | GCGCCGTCTCGCTCGCATTTT  GGATCCAGTAAAGCAAAGTATC |

*HSc70a* means primers design to amplify different alleles region of *HSc70* gene, *HSc70*p750 means forward primer designed to amplify -750 bp from ATG *Hsc70* code region; *HSc70*_A1 means reverse primer designed to amplify *HSc70* code region from A1 allele; *HSc70*_A3 means reverse primer designed to amplify *HSc70* code region from A3 allele; *HSc70*_A4 means reverse primer designed to amplify *HSc70* code region from A4 allele; *HSc70*_Common means reverse primer designed to amplify *HSc70* code region from common region to discriminate between A2. *HSc70*p1000 means primers designed to amplify -1000 bp from ATG *HSc70* code region, *HSc70*cd means primers designed to amplify *HSc70* code region, *HSc70*p1000_GB means primers designed to amplify -1000bp promoter with bar codes specific to Golden Braid strategy, *HSc70*cd_GB means primers designed to amplify *HSc70* code region with bar-codes specific to Golden Braid strategy.

**Table S2**. Primer sequences and probe used in qRT-PCR

| Name | Forward | Reverse | Probe |
| --- | --- | --- | --- |
| *HSc70* | ATGAAGCTTTGGCCTTTCAA | TTTGTAATTGACAACAATCATAGGC | UPL62: ACCTGCTG |
| *EF1α* | CTTGACGCTCTTGACCAGATT | GAAGACGGAGGGGTTTGTCT | UPL117: AGCCCAAG |

**Table S3**. Nodal cutting results of *HSc70* overexpression lines. Fresh weight (FW), dry weight (DW).

| Trait measured | Line 33 (g) | Line 48 (g) | Line 56 (g) | WT (g) |
| --- | --- | --- | --- | --- |
| FW at 20⁰C | 3.29±0.43^a^ | 3.58±0.55^a^ | 3.73±0.37^a^ | 3.93±0.33^a^ |
| FW at 28⁰C | 1.55±0.28^b^ | 1.96±0.32^b^ | 2.03±0.41^b^ | 1.09±0.20^a^ |
| DW at 20⁰C | 0.46±0.05^a^ | 0.59±0.09^a^ | 0.62±0.06^a^ | 0.63±0.05^a^ |
| DW at 28⁰C | 0.25±0.03^b^ | 0.27±0.05^b^ | 0.28±0.05^b^ | 0.15±0.03^a^ |

Data are presented as mean ± standard error and different letter indicate statistical differences between lines (Fisher’s, p<0.05).

171 HSc70_A2 ATGGCCGGAAAAGGTGAAGGACCGGCGATCGGAATTGATCTCGGAACGACGTATTCGTGT

99 HSc70_A3 ATGGCCGGAAAAGGTGAAGGACCGGCGATCGGAATTGATCTCGGAACGACGTATTCGTGT

171 HSc70_A1 ATGGCCGGAAAAGGTGAAGGACCGGCGATCGGAATTGATCTCGGAACAACGTATTCGTGT

99 HSc70_A4 ATGGCCGGAAAAGGTGAAGGACCGGCGATCGGAATTGATCTCGGAACGACGTATTCGTGT

*********************************************** ************

171 HSc70_A2 GTAGGTGTATGGCAGCATGATCGAGTTGAGATTATTGCGAATGATCAAGGGAACAGGACT

99 HSc70_A3 GTAGGTGTATGGCAGCATGACCGGGTTGAGATTATTGCGAATGATCAAGGGAACAGGACT

171 HSc70_A1 GTAGGTGTATGGCAGCATGATCGAGTTGAGATTATTGCTAATGATCAAGGGAACAGGACT

99 HSc70_A4 GTAGGTGTATGGCAGCATGATCGAGTTGAGATTATTGCGAATGATCAAGGGAACAGGACT

******************** ** ************** *********************

171 HSc70_A2 ACGCCGTCGTATGTGGCGTTTACTGATACGGAGCGATTGATTGGAGATGCTGCTAAGAAT

99 HSc70_A3 ACGCCGTCGTATGTGGCGTTTACTGATACGGAGCGATTGATTGGAGATGCTGCTAAGAAT

171 HSc70_A1 ACGCCGTCGTATGTGGCGTTTACTGATACGGAGCGATTGATTGGAGATGCTGCTAAGAAT

99 HSc70_A4 ACGCCGTCGTATGTGGCGTTTACTGATACGGAGCGATTGATTGGAGATGCTGCTAAGAAT

************************************************************

171 HSc70_A2 CAAGTTGCTATGAACCCTACCAACACTGTTTTCGGTAAGTTCTGGAAATTCCGTGCTTTG

99 HSc70_A3 CAAGTTGCTATGAACCCTACCAACACTGTTTTCGGTAAGTTCTGGAAATTCCGTGCTTTG

171 HSc70_A1 CAAGTTGCTATGAACCCTACTAACACTGTTTTCGGTAAGTTCTGGAGATTCATGA-----

99 HSc70_A4 CAAGTTGCTATGAACCCTACCAACACTGTTTTCGGTAAGTTCTGGAAATTCCGTGCTTTG

******************** ************************* ****

171 HSc70_A2 TGATTGGTATTTGTTATTTATGATTCTCTCTTTTTGTTCAGTATTCGAACTAAGATTTTG

99 HSc70_A3 TGATTGGTATTTGTTATTTATGATTCTCTCTCTTTGTTCAGTATTCGAACTAAGATTTTG

171 HSc70_A1 ------------------------------TTCTCGTTCAGTATTCGAATTAGGATTTTG

99 HSc70_A4 TGATTGGTATTTGTTATTCATGATTCTCTCTTTTTGTTCAGTATTCGAACTAGGATTTTG

* * ************** ** *******

171 HSc70_A2 AGTTTATGTTCAGTATTTGAGCAAGTATTATGAGTTTATGTTCAGTATTTGAACTTATGT

99 HSc70_A3 AGTTTATGTTCAGTATTTGAGCAAGTATTATGAGTTTAT-------------------GT

171 HSc70_A1 AGTTTATGTTCAGTATTTGAGCTAGGATTTTGAGCTTATGTTTAGTATATTGG-------

99 HSc70_A4 AGTTTATGTTCAGTATTTGAGCTAGGATTTTGAGCTTATGTTTAGTATATATT-------

********************** ** *** **** ****

171 HSc70_A2 TTGGTATATTGGAGCAAGGATTTTGAGTTTATGTTTAGTAT---------------TGGA

99 HSc70_A3 TTGGTATATTGGAGCAAGGATTTTGAGTTTATGTTTAGTAT---------------TGGA

171 HSc70_A1 ---------------------------------------------AGCAAGGATTTGAGT

99 HSc70_A4 ----------GGAGCAAGGATTTTGAGTTTATGTTTAGTATTGGAGCTAGGATTTTGAGT

*

171 HSc70_A2 GCTAGGATTTTGAATTTGATTAGGTTCTCGATTAATCATTTATACCTATTAAGCTAGATT

99 HSc70_A3 GCTAGGATTTTGAATTTGATTAGGTTCTCGATTAATCATTTATACCTATTAAGCTAGATT

171 HSc70_A1 TCATGAATTTTGAATTTGATTAGGTTCTCGATTAGTCATTTATACCTATTAAGCTAGATT

99 HSc70_A4 TTATGAATTTTGAATTTGATTAGGTTCTCGATTAATCATTTACAACTGTTAAGCTAGATT

* **************************** ******* * ** ************

171 HSc70_A2 TTTGAAAACATAAATTTAGTGCAAGTAAAACTGTATCTCTTCTCTGTTTCATCAATTCAT

99 HSc70_A3 TTTGAAAACATAAATTTTGTGCAAGTTAAACTGTATCTCTTCTCTGTTTCATCAATTCAT

171 HSc70_A1 TTCGAAAACATAAATTTAGGGTGAGTAAAACTGTATATATTCCCTGTTTCATCAATTCAT

99 HSc70_A4 TTCGAAAACATAAATTTAGGGTAAGTAAAACTGTATCTCTGCCCTGTTTCATCAGTTCAT

** ************** * * *** ********* * * * *********** *****

171 HSc70_A2 CAAGCTTATTGGTCCTGATTTGTATCTGCATTTTCAACTTATGGTCTGTTGATCATAAGT

99 HSc70_A3 CAAGCTTATTGGTCCTGATTTGTATCTGCATTTTCAACTTATGGTCTGTTGATCATAAGT

171 HSc70_A1 CAAGCT---AGGTCCTGATTTGTATCTGCATTTTCAACTTATGGTCTGTTGATCATAAGT

99 HSc70_A4 CAAGCTTATTGGTCCTGATTTGTATCTACATTTTCAACTTAGGGTCTGTTGATCATAACT

****** ***************** ************* **************** *

171 HSc70_A2 AATCGTAGCTCTGCCCTGGTTTTGTGCTAGTTTTTTATATAGTACCATAACAATGTTGAG

99 HSc70_A3 AATTGTAGCTCTGCCCTGGTTTTGTGCTAGTTTTTTATATAGTACCATAACAATGTAGAG

171 HSc70_A1 AATTGTAGCTCTGCCCTGGTTTTTTGTTAGTTTTTTATATAGTACCATAACAATGAAGAG

99 HSc70_A4 AATCGTAGCTCTGCCCTGGTTTTTTGTTAGT---TTATATAGTACCATAACAATGAAGAG

*** ******************* ** **** ********************* ***

171 HSc70_A2 TGTTCAATTTAAGCTTATAAGAAGTGGAATCTGGCGAATTTGTCCAGCTTAACCATGTTA

99 HSc70_A3 TGTTCAATTTAAGCTTATAAGAAGTGGAATCTGGCGAATTTGTCCAGCTTAACCATGTTA

171 HSc70_A1 TGTTCAATTTAAGTTTAT-AGAAGTGGAATCTGGCGAATTTGTCCAGCTTAACCATGTTA

99 HSc70_A4 TGTTCAATTTAAGTTTAT-TGAAGTGGAATCTGTCGAATTCGTCCAGCTTAACCATGTTA

************* **** ************* ****** *******************

171 HSc70_A2 GGCTAAAATTATCAAAGATAATAATTTCTTGTTTTTGTATGCTGTATTTACTCTGCTGTA

99 HSc70_A3 GGCTAAAATTATCAAAGATAATAATTTCTTGTTTTTGTATGCTGTATTTACTCTGCTGTA

171 HSc70_A1 GGCTAAAATTATCAAAGATAATAATTTCTTGTTTTTGTATGCTGTATTTACTCTGCTGTA

99 HSc70_A4 GGCTAAAACTATAAACGATAATAAATTCTTGTTTTTGTATGCTATATTTACTCCGCTGTA

******** *** ** ******** ****************** ********* ******

171 HSc70_A2 GCCACTGATAAATTTTTAGCTTACCCAAATTATCTGTTCAATTGAAGTTTCAAGTAAGAA

99 HSc70_A3 GCCACTGATAAATTTTTAGCTTACCCAAATTATCTGTTCAATTGAAGTTTCAAGTAAGAA

171 HSc70_A1 GCCACTGATAAATTTTTAGCTTACCCAAATTATCTGTTCAATTGAAGTTTCAAGTAAGAA

99 HSc70_A4 GCCACTGACAAATTTTTAGCTTACCCAAATTATCGGTTCAATTGAAGTTTCAAGTAAGAA

******** ************************* *************************

171 HSc70_A2 AGAAAGTAATTATGTTTGAATTCATGCTGAAATCTTTTATAATTTGATTGCAGATGCGAA

99 HSc70_A3 AGAAAGTAACTATGTTTGAATTCATGCTGAAATCTTTTATAATTTGATTGCAGATGCGAA

171 HSc70_A1 AGAAAGTAATTATGTTTGAATTCATGCTGAAATCTTTTATAATTTGATTGCAGATGCGAA

99 HSc70_A4 AGAAAGTAATTATGTTTGAATTCATGCTGAAATCTTTTATAATTTGATTGCAGATGCGAA

********* **************************************************

171 HSc70_A2 GCGATTAATTGGTAGGAGATTCAGTGACCCATCAGTACAGAGTGACATGAAGCTTTGGCC

99 HSc70_A3 GCGATTAATTGGTAGGAGATTCAGTGACCCATCAGTACAGAGTGACATGAAGCTTTGGCC

171 HSc70_A1 GCGATTAATTGGTAGGAGATTCAGTGACCCATCAGTACAGAGTGACATGAAGCTTTGGCC

99 HSc70_A4 GCGATTAATTGGTAGGAGATTCAGTGACCCATCAGTACAGAGTGACATGAAGCTTTGGCC

************************************************************

171 HSc70_A2 TTTCAAGGTCATCCCAGGACCTGCTGACAAGCCTATGATTGTTGTCAATTACAAAGGTGA

99 HSc70_A3 TTTCAAGGTCATCCCAGGACCTGCTGACAAGCCTATGATTGTTGTCAATTACAAAGGTGA

171 HSc70_A1 TTTCAAGGTCATCCCAGGACCTGCTGACAAGCCTATGATTGTTGTCAATTACAAAGGTGA

99 HSc70_A4 TTTCAAGGTCATCCCAGGACCTGCTGACAAGCCTATGATTGTTGTCAATTACAAAGGTGA

************************************************************

171 HSc70_A2 AGAAAAAGAGTTCTCCGCAGAAGAAATCTCCTCCATGGTTCTTATTAAGATGAAGGAGAT

99 HSc70_A3 AGAAAAAGAGTTCTCCGCAGAAGAAATCTCCTCCATGGTTCTTATTAAGATGAAGGAGAT

171 HSc70_A1 AGAAAAAGAGTTCTCCGCAGAAGAAATCTCCTCCATGGTTCTTATTAAGATGAAGGAGAT

99 HSc70_A4 AGAAAAAGAGTTCTCCGCAGAAGAAATCTCCTCCATGGTTCTTATTAAGATGAAGGAGAT

************************************************************

171 HSc70_A2 TGCTGAGGCTTTTCTTGGAATAACAATAAAGAATGCTGTTGTTACTGTGCCTGCTTACTT

99 HSc70_A3 TGCTGAGGCTTTTCTTGGAATAACAATAAAGAATGCTGTTGTTACTGTGCCTGCTTACTT

171 HSc70_A1 TGCTGAGGCTTTTCTTGGAATAACAATAAAGAATGCTGTTGTTACTGTGCCTGCTTACTT

99 HSc70_A4 TGCTGAGGCTTTTCTTGGAATAACAATAAAGAATGCTGTTGTTACTGTGCCTGCTTACTT

************************************************************

171 HSc70_A2 CAATGACTCTCAACGTCAGGCTACTAAGGATGCTGGAACTATTTCTGGGCTCAATGTTAT

99 HSc70_A3 CAATGACTCTCAACGTCAGGCTACTAAGGATGCTGGAACTATTTCTGGGCTCAATGTTAT

171 HSc70_A1 CAATGACTCTCAACGTCAGGCTACTAAGGATGCTGGAACTATTTCTGGGCTCAATGTTAT

99 HSc70_A4 CAATGACTCTCAACGTCAGGCTACTAAGGATGCTGGAACTATTTCTGGGCTCAATGTTAT

************************************************************

171 HSc70_A2 GCGTATTATCAACGAGCCTACTGCTGCTGCAATTGCATATGGACTTGATAAGAAATCAAG

99 HSc70_A3 GCGTATTATCAACGAGCCTACTGCTGCTGCAATTGCATATGGACTTGATAAGAAATCAAG

171 HSc70_A1 GCGTATTATCAACGAGCCTACTGCTGCTGCAATTGCATATGGACTTGATAAGAAATCAAG

99 HSc70_A4 GCGTATTATCAACGAGCCTACTGCTGCTGCAATTGCATATGGACTTGATAAGAAATCAAG

************************************************************

171 HSc70_A2 CAGTACAGGGGAGAAGACTGTGCTTATTTTTGACTTGGGTGGTGGTACATTTGATGTCTC

99 HSc70_A3 CAGTACAGGGGAGAAGACTGTGCTTATTTTTGACTTGGGTGGTGGTACATTTGATGTCTC

171 HSc70_A1 CAGTACAGGGGAGAAGACTGTGCTTATTTTTGACTTGGGTGGTGGTACATTTGATGTCTC

99 HSc70_A4 CAGTACAGGGGAGAAGACTGTGCTTATTTTTGACTTGGGTGGTGGTACATTTGATGTCTC

************************************************************

171 HSc70_A2 ACTACTTACCATTGAAGAAGGTATTTTTGAAGTGAAAGCCACTGCTGGTGATACTCATCT

99 HSc70_A3 ACTACTTACCATTGAAGAAGGTATTTTTGAAGTGAAAGCCACTGCTGGTGATACTCATCT

171 HSc70_A1 ACTACTTACCATTGAAGAAGGTATTTTTGAAGTGAAAGCCACTGCTGGTGATACTCATCT

99 HSc70_A4 ACTACTTACCATTGAAGAAGGTATTTTTGAAGTGAAAGCCACTGCTGGTGATACTCATCT

************************************************************

171 HSc70_A2 TGGTGGAGAAGATTTTGACAACAGGATGGTTAATCACTTTGTTCAAGAATTCAAAAGGAA

99 HSc70_A3 TGGTGGAGAAGATTTTGACAACAGGATGGTTAATCACTTTGTTCAAGAATTCAAGAGGAA

171 HSc70_A1 TGGTGGAGAAGATTTTGACAACAGGATGGTTAATCACTTTGTTCAAGAATTCAAGAGGAA

99 HSc70_A4 TGGTGGAGAAGATTTTGACAACAGGATGGTTAATCACTTTGTTCAAGAATTCAAGAGGAA

****************************************************** *****

171 HSc70_A2 GCACAAAAAGGACATCAGTGGGAATCCTAGAGCTCTTAGGAGATTAAGAACTGCATGTGA

99 HSc70_A3 GCACAAGAAAGACATTAGTGGGAATCCTAGAGCTCTTAGGAGATTAAGAACTGCATGTGA

171 HSc70_A1 GCACAAAAAGGACATCAGTGGGAATCCTAGAGCTCTTAGGAGATTAAGAACTGCATGTGA

99 HSc70_A4 GCACAAAAAGGACATCAGTGGGAATCCTAGAGCTCTTAGGAGATTAAGAACTGCATGTGA

****** ** ***** ********************************************

171 HSc70_A2 AAGAGCTAAGAGAACACTCTCATCCACTGCTCAGACAACTATTGAAATTGATTCTCTTTA

99 HSc70_A3 AAGAGCTAAGAGAACACTCTCATCCACTGCTCAGACAACTATTGAAATTGATTCTCTTTA

171 HSc70_A1 AAGAGCTAAGAGAACACTCTCATCCACTGCTCAGACAACTATTGAAATTGATTCTCTTTA

99 HSc70_A4 AAGAGCTAAGAGAACACTCTCATCCACTGCTCAGACAACTATTGAAATTGATTCTCTTTA

************************************************************

171 HSc70_A2 TGAGGGAATTGATTTCTACACGACGATTACTCGTGCTCGGTTTGAGGAGCTCAACATGGA

99 HSc70_A3 TGAGGGAATTGACTTCTACACGACGATTACTCGTGCTCGGTTTGAGGAGCTCAACATGGA

171 HSc70_A1 TGAGGGAATTGATTTCTACACGACGATTACTCGTGCTCGGTTTGAGGAGCTCAACATGGA

99 HSc70_A4 TGAGGGAATTGATTTCTACACGACGATTACTCGTGCTCGGTTTGAGGAGCTCAACATGGA

************ ***********************************************

171 HSc70_A2 TCTTTTCAGGAAATGTATGGAGCCTGTTGAGAAGTGTTTGAGGGATGCTAAAATTGACAA

99 HSc70_A3 TCTTTTCAGGAAATGTATGGAGCCTGTTGAGAAGTGTTTGAGGGATGCTAAAATTGACAA

171 HSc70_A1 TCTTTTCAGGAAATGTATGGAGCCTGTTGAGAAGTGTTTGAGGGATGCTAAAATTGACAA

99 HSc70_A4 TCTTTTCAGGAAATGTATGGAGCCTGTTGAGAAGTGTTTGAGGGATGCTAAAATTGACAA

************************************************************

171 HSc70_A2 GAGTGGTGTTCATGATATTGTTCTTGTTGGTGGCTCTACTCGAATTCCAAAGGTGCAACA

99 HSc70_A3 GAGTGGTGTTCATGATATTGTTCTTGTTGGTGGCTCTACTAGAATTCCAAAGGTGCAACA

171 HSc70_A1 GAGTGGTGTTCATGATATTGTTCTTGTTGGTGGCTCTACTCGAATTCCAAAGGTGCAACA

99 HSc70_A4 GAGTGGTGTTCATGATATTGTTCTTGTTGGTGGCTCTACTCGAATTCCAAAGGTGCAACA

**************************************** *******************

171 HSc70_A2 GTTGTTGCAGGACTTCTTTAACGGGAAGGAACTCTGTAAGAGCATCAACCCGGACGAGGC

99 HSc70_A3 GTTGTTGCAGGACTTCTTTAACGGGAAGGAACTCTGTAAGAGCATCAACCCGGATGAGGC

171 HSc70_A1 GTTGTTGCAGGACTTCTTTAACGGGAAGGAACTCTGTAAGAGCATCAACCCGGACGAGGC

99 HSc70_A4 GTTGTTGCAGGACTTCTTTAACGGGAAGGAACTCTGTAAGAGCATCAACCCGGACGAGGC

****************************************************** *****

171 HSc70_A2 TGTTGCGTATGGAGCTGCTGTGCAAGCTGCAATCTTGAGTGGTGAAGGGAATGAAAAAGT

99 HSc70_A3 TGTTGCGTATGGAGCTGCTGTGCAAGCTGCAATCTTGAGTGGTGAAGGGAATGAAAAAGT

171 HSc70_A1 TGTTGCGTATGGAGCTGCTGTGCAAGCTGCAATCTTGAGTGGTGAAGGGAATGAAAAAGT

99 HSc70_A4 TGTTGCGTATGGAGCTGCTGTGCAAGCTGCAATCTTGAGTGGTGAAGGGAATGAAAAAGT

************************************************************

171 HSc70_A2 TCAGGACCTTTTGCTGTTGGATGTTACACCTCTTTCCCTTGGTCTGGAGACTGCTGGAGG

99 HSc70_A3 TCAGGACCTTTTGCTGTTGGATGTTACACCTCTTTCCCTTGGTCTGGAGACTGCTGGAGG

171 HSc70_A1 TCAGGACCTTTTGCTGTTGGATGTTACACCTCTTTCCCTTGGTCTGGAGACTGCTGGAGG

99 HSc70_A4 TCAGGACCTTTTGCTGTTGGATGTTACACCTCTTTCCCTTGGTCTGGAGACTGCTGGAGG

************************************************************

171 HSc70_A2 TGTTATGACCACCTTGATTCCAAGGAACACCACGATTCCGACTAAGAAAGAGCAGGTGTT

99 HSc70_A3 TGTTATGACCACCTTGATTCCAAGGAACACCACCATACCGACTAAGAAAGAGCAGGTGTT

171 HSc70_A1 TGTTATGACCACCTTGATTCCAAGGAACACCACGATTCCGACTAAGAAAGAGCAGGTGTT

99 HSc70_A4 TGTTATGACCACCTTGATTCCAAGGAACACCACGATTCCGACTAAGAAAGAGCAGGTGTT

********************************* ** ***********************

171 HSc70_A2 TTCGACCTACTCCGACAATCAACCTGGTGTGTTGATTCAAGTTTATGAAGGTGAAAGAGC

99 HSc70_A3 TTCGACCTACTCCGACAATCAACCTGGTGTGTTGATTCAAGTTTATGAAGGTGAAAGAGC

171 HSc70_A1 TTCGACCTACTCCGACAATCAACCTGGTGTGTTGATTCAAGTTTATGAAGGTGAAAGAGC

99 HSc70_A4 TTCGACCTACTCCGACAATCAACCTGGTGTGTTGATTCAAGTTTATGAAGGTGAAAGAGC

************************************************************

171 HSc70_A2 CAGGACTAGAGATAACAACTTGCTGGGGAAATTTGAACTCACTGGCATTCCTCCAGCACC

99 HSc70_A3 CAGGACTAGAGATAACAACTTGCTAGGGAAATTTGAACTCACTGGCATTCCTCCAGCACC

171 HSc70_A1 CAGGACTAGAGATAACAACTTGCTGGGGAAATTTGAACTCACTGGCATTCCTCCAGCACC

99 HSc70_A4 CAGGACTAGAGATAACAACTTGCTGGGGAAATTTGAACTCACTGGCATTCCTCCAGCACC

************************ ***********************************

171 HSc70_A2 TAGAGGTGTCCCCCAAATTACTGTATGCTTTGACATTGACGCAAATGGTATTTTAAATGT

99 HSc70_A3 TAGAGGTGTCCCCCAAATTACTGTATGCTTTGACATTGACGCAAATGGTATTTTAAATGT

171 HSc70_A1 TAGAGGTGTCCCCCAAATTACTGTATGCTTTGACATTGACGCAAATGGTATTTTAAATGT

99 HSc70_A4 TAGAGGTGTCCCCCAAATTACTGTATGCTTTGACATTGACGCAAATGGTATTTTAAATGT

************************************************************

171 HSc70_A2 CTCTGCTGAGGATAAAACCACTGGTCAGAAAAACAAGATTACCATCACCAATGACAAAGG

99 HSc70_A3 CTCTGCTGAGGATAAAACAACTGGTCAGAAAAACAAGATTACCATCACCAATGACAAAGG

171 HSc70_A1 CTCTGCTGAGGATAAAACCACTGGTCAGAAAAACAAGATTACCATCACCAATGACAAAGG

99 HSc70_A4 CTCTGCTGAGGATAAAACCACTGGTCAGAAAAACAAGATTACCATCACCAATGACAAAGG

****************** *****************************************

171 HSc70_A2 AAGACTATCTAAAGATGAGATTGAGAAAATGGTTCAAGAAGCTGAGAGGTACAAAGCTGA

99 HSc70_A3 AAGACTATCTAAAGATGAGATTGAGAAAATGGTTCAAGAAGCTGAGAGGTACAAAGCTGA

171 HSc70_A1 AAGACTATCTAAAGATGAGATTGAGAAAATGGTTCAAGAAGCTGAGAGGTACAAAGCTGA

99 HSc70_A4 AAGACTATCTAAAGATGAGATTGAGAAAATGGTTCAAGAAGCTGAGAGGTACAAAGCTGA

************************************************************

171 HSc70_A2 AGACGAAGAGCACAAGAAGAAAGTGGAAGCCAAGAATGCCTTGGAAAATTATGCCTACAA

99 HSc70_A3 AGACGAAGAGCACAAGAAGAAAGTGGAAGCCAAGAATGCCTTGGAAAATTATGCCTACAA

171 HSc70_A1 AGACGAAGAGCACAAGAAGAAAGTGGAAGCCAAGAATGCCTTGGAAAATTATGCCTACAA

99 HSc70_A4 AGACGAAGAGCACAAGAAGAAAGTGGAAGCCAAGAATGCCTTGGAAAATTATGCCTACAA

************************************************************

171 HSc70_A2 CATGAGGAACACCATCAAGGATGATAAGATCGCTTCGAAGCTTTCTCCTGAGGAGAAGCA

99 HSc70_A3 CATGAGGAACACCATCAAGGATGATAAGATCGCTTCGAAGCTTTCTCCTGAGGAGAAGCA

171 HSc70_A1 CATGAGGAACACCATCAAGGATGATAAGATCGCTTCGAAGCTTTCTCCTGAGGAGAAGCA

99 HSc70_A4 CATGAGGAACACCATCAAGGATGATAAGATCGCTTCGAAGCTTTCTCCTGAGGAGAAGCA

************************************************************

171 HSc70_A2 GAAGATAGAAGATTCTGTTGAGCAGGCAATTCAGTGGCTTGATGGGAATCAACTCGCGGA

99 HSc70_A3 GAAGATAGAAGATTCGGTTGAGCAGGCGATTCAGTGGCTTGATGGGAATCAACTCGCGGA

171 HSc70_A1 GAAGATAGAAGATTCTGTTGAGCAGGCAATTCAGTGGCTTGATGGGAATCAACTCGCGGA

99 HSc70_A4 GAAGATAGAAGATTCTGTTGAGCAGGCAATTCAGTGGCTTGATGGGAATCAACTCGCGGA

*************** *********** ********************************

171 HSc70_A2 GGCTGATGAGTTTGAGGACAAGATGAAGGAGCTTGAGAGCATATGCAACCCCATTATCGC

99 HSc70_A3 GGCTGATGAGTTTGAGGACAAGATGAAGGAGCTTGAGAGCATATGCAACCCCATTATCGC

171 HSc70_A1 GGCTGATGAGTTTGAGGACAAGATGAAGGAGCTTGAGAGCATATGCAACCCCATTATCGC

99 HSc70_A4 GGCTGATGAGTTTGAGGACAAGATGAAGGAGCTTGAGAGCATATGCAACCCCATTATCGC

************************************************************

171 HSc70_A2 GAAGATGTACCAGGGTGGTG---------CTGACATGGCTGGTGGCATGGATGAAGATGG

99 HSc70_A3 GAAGATGTACCAGGGTGGTGCTGGTGGTCCTGACATGGCTGGTGGCATGGATGAAGATGG

171 HSc70_A1 GAAGATGTACCAGGGTGGTG---------CTGACATGGCTGGTGGCATGGATGAAGATGG

99 HSc70_A4 GAAGATGTACCAGGGTGGTG---------CTGACATGGCTGGTGGCATGGATGAAGATGG

******************** *******************************

171 HSc70_A2 CCCTTCTGCCGGTGCTAGCGGTGCTGGTGCAGGCCCTAAGATTGAAGAAGTTGATTAA

99 HSc70_A3 CCCTTCAGCCGGTGCTAGCGGTGCTGGTGCAGGCCCTAAGATTGAAGAAGTTGATTAA

171 HSc70_A1 CCCTTCTGCCGGTGCTAGCGGTGCTGGTGCAGGCCCTAA-------------------

99 HSc70_A4 CCCTTCTGCCGGTGCTAGCGGTGCTGGTGCAGGCCCTAA-------------------

****** ********************************

**Figure S1.** Clustal Omega alignment of the genomic DNA sequences of HSc70 alleles A1, A2, A3 and A4 isolated from parents of the 06H1 population (HB171(13) and 99FT1b5). Intron is indicated by blue text. Quantitative PCR primers and probe binding sites are highlighted in grey. Reverse primers used to amplify promoter regions: HSC70PA1R CTGAACGAGAATCATGAATCT; HSC70PCOMMONR AGATGCGAAGCGATTAATTGGT; HSC70PA3R TATACCAAACATAAACTCAT; HSC70PA4R TCCTAGCTCCAATACTAAACA

A3 KMKELESICNPIIAKMYQGGAGGPDMAGGMDEDGPSAGASGAGAGPKIEEVD

A1 KMKELESICNPIIAKMYQGGAGGPDMAGGMDEDGPSAGASGAGAGPX------

A4 KMKELESICNPIIAKMYQGGAGGPDMAGGMDEDGPSAGASGAGAGPX------

A2 KMKELESICNPIIAKMYQGGAGGPDMAGGMDEDGPSAGASGAGAGPKIEEVD

**Figure S2**. CLUSTAL O (1.2.1) multiple sequence alignment of the predicted *HSc70* amino acid sequences from the C-terminal region of 4 alleles.

**(a)**


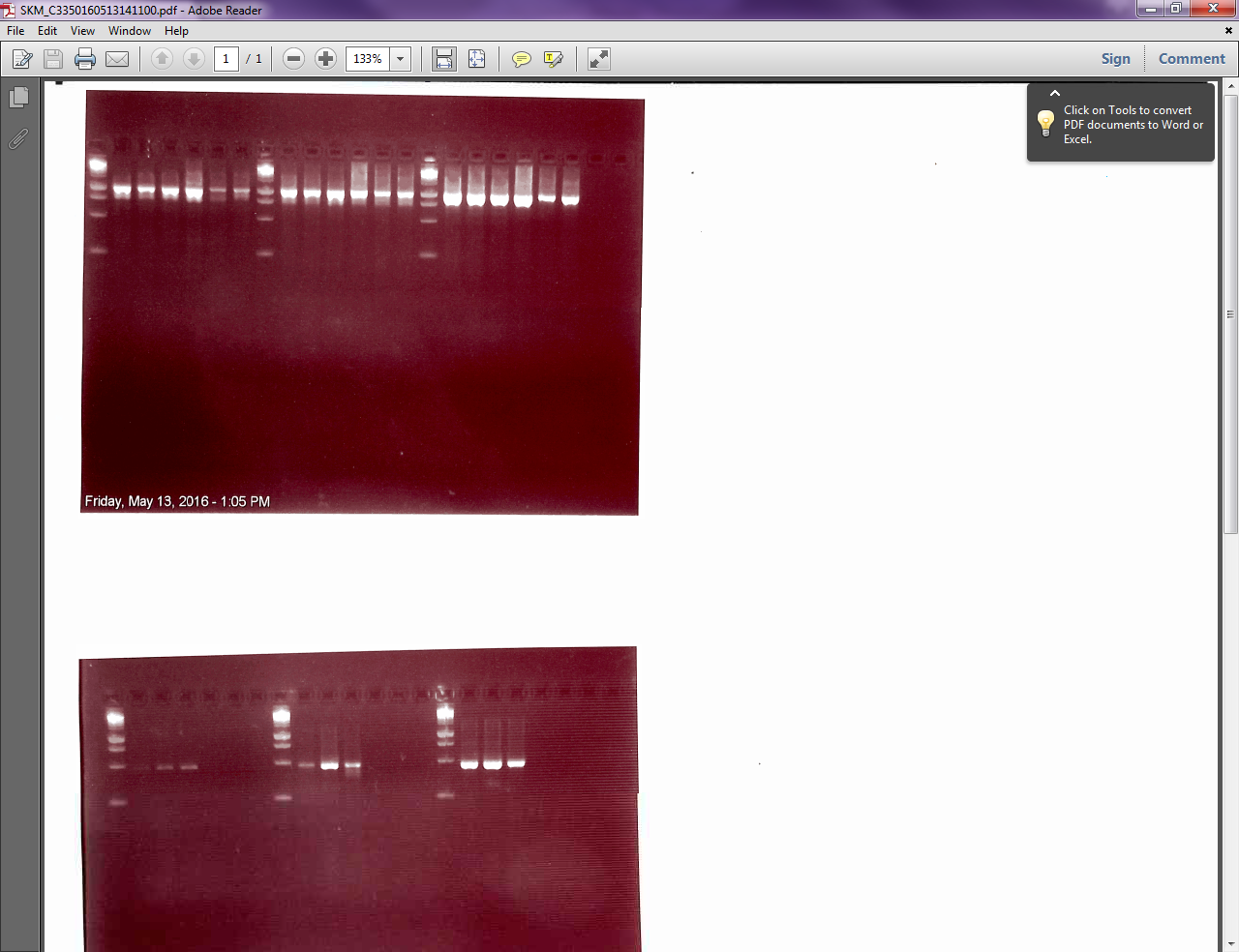

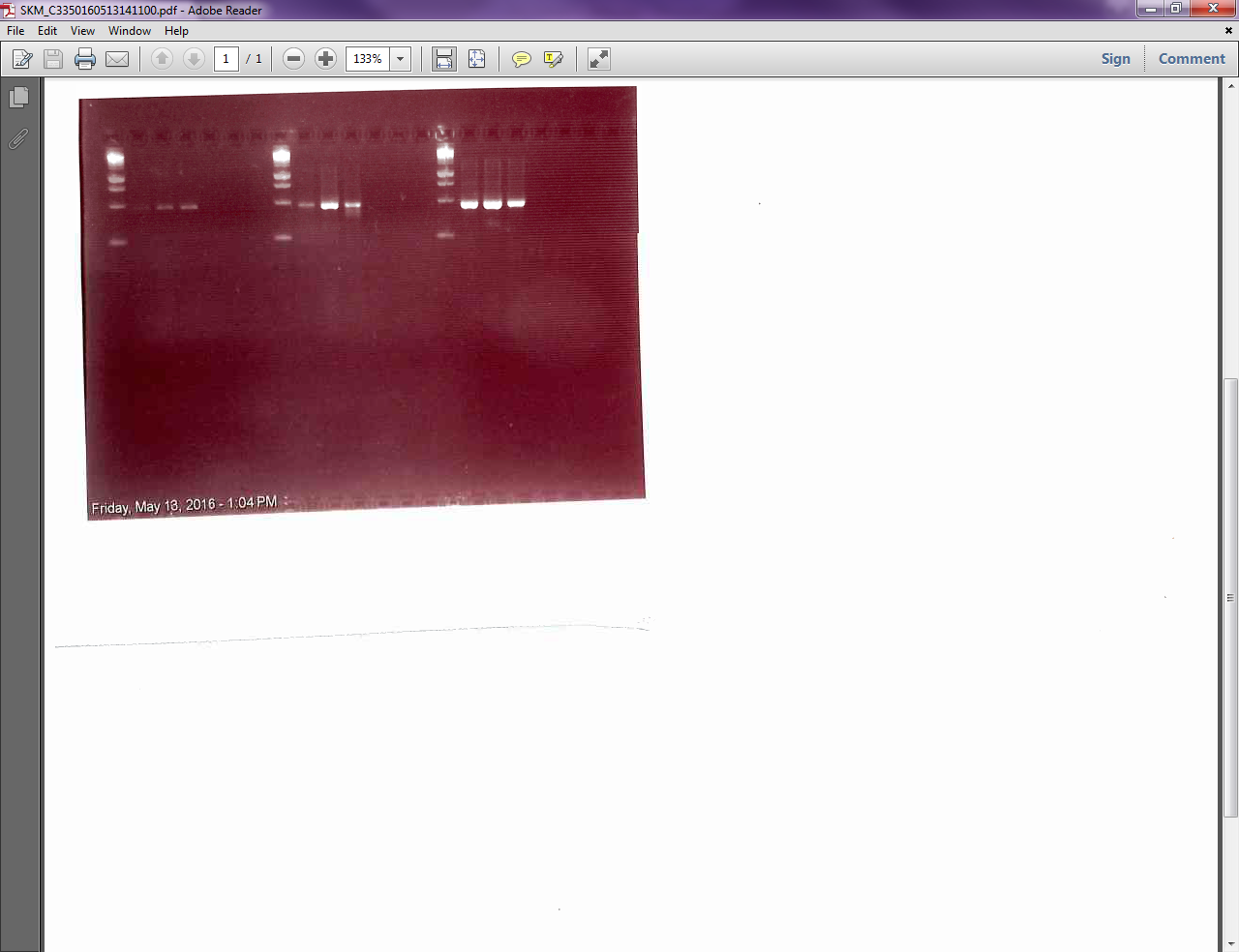


*HSc70*

*EF1α*

HT

HS

HT

HS

HT

HS

C L H


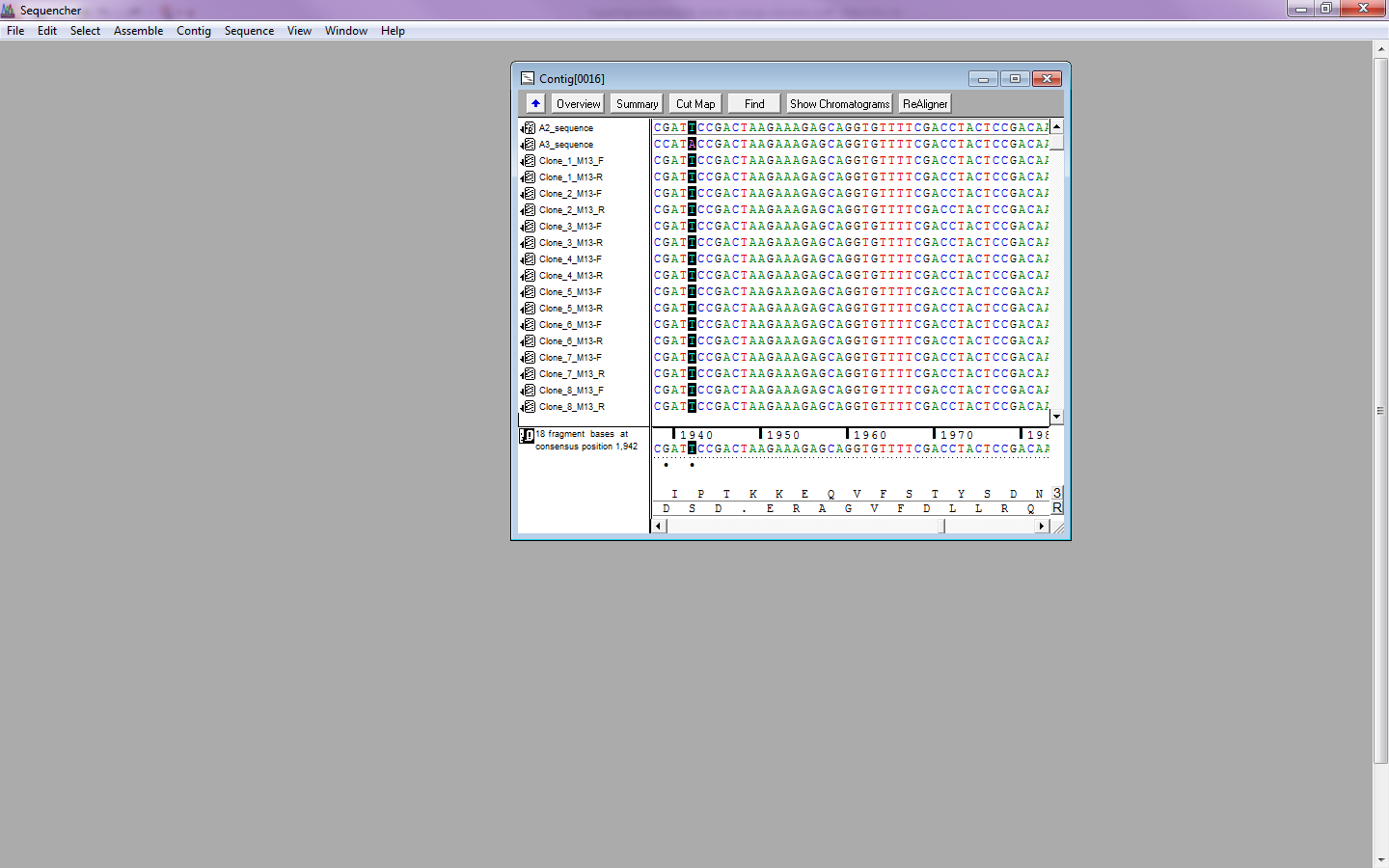


C L H

C L H

C L H

C L H

C L H

**(b)**

**Figure S3.** Expression level of *HSc70* by semi-quantitative RT-PCR. (a) Bands of semi-quantitative RT-PCR in agarose gel (1%). HT means heat tolerante genotype; HS means heat sensitive genotype; C means control, L means 4h at 20⁰C and H measn 4h at 28⁰C. (b) Sequence analysis of clone products with *HSc70*-A2 sequence and *HSc70*-A3 sequence as model, where all transcripts show GATT region as A2.


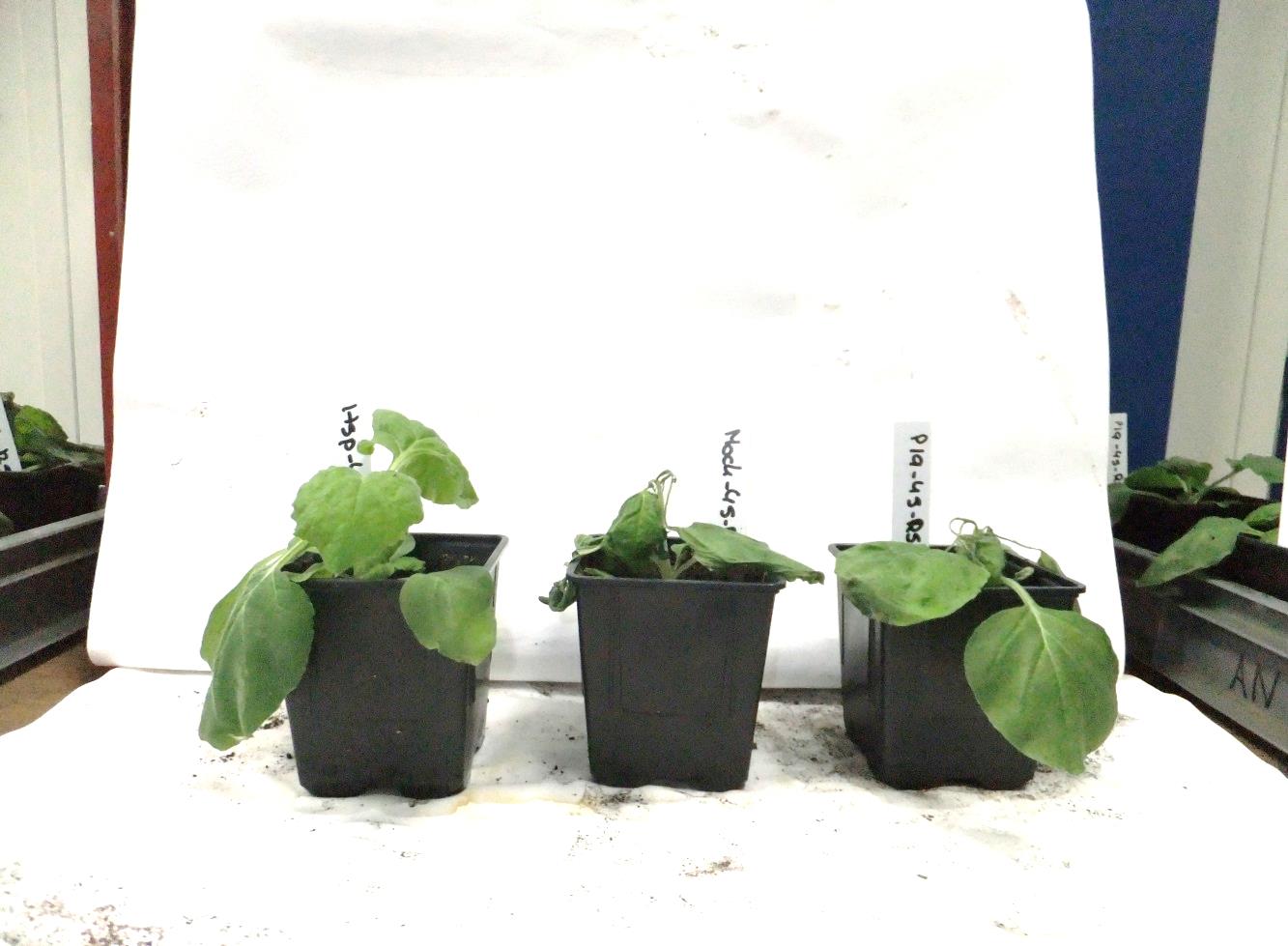


***HSc70***

**Mock**

**Visual assessment**

**Figure S4**. Phenotype of *HSc70* agro-infiltrated and Mock inoculated plants of *Nicotiana benthamiana* after 24h at 45⁰C.


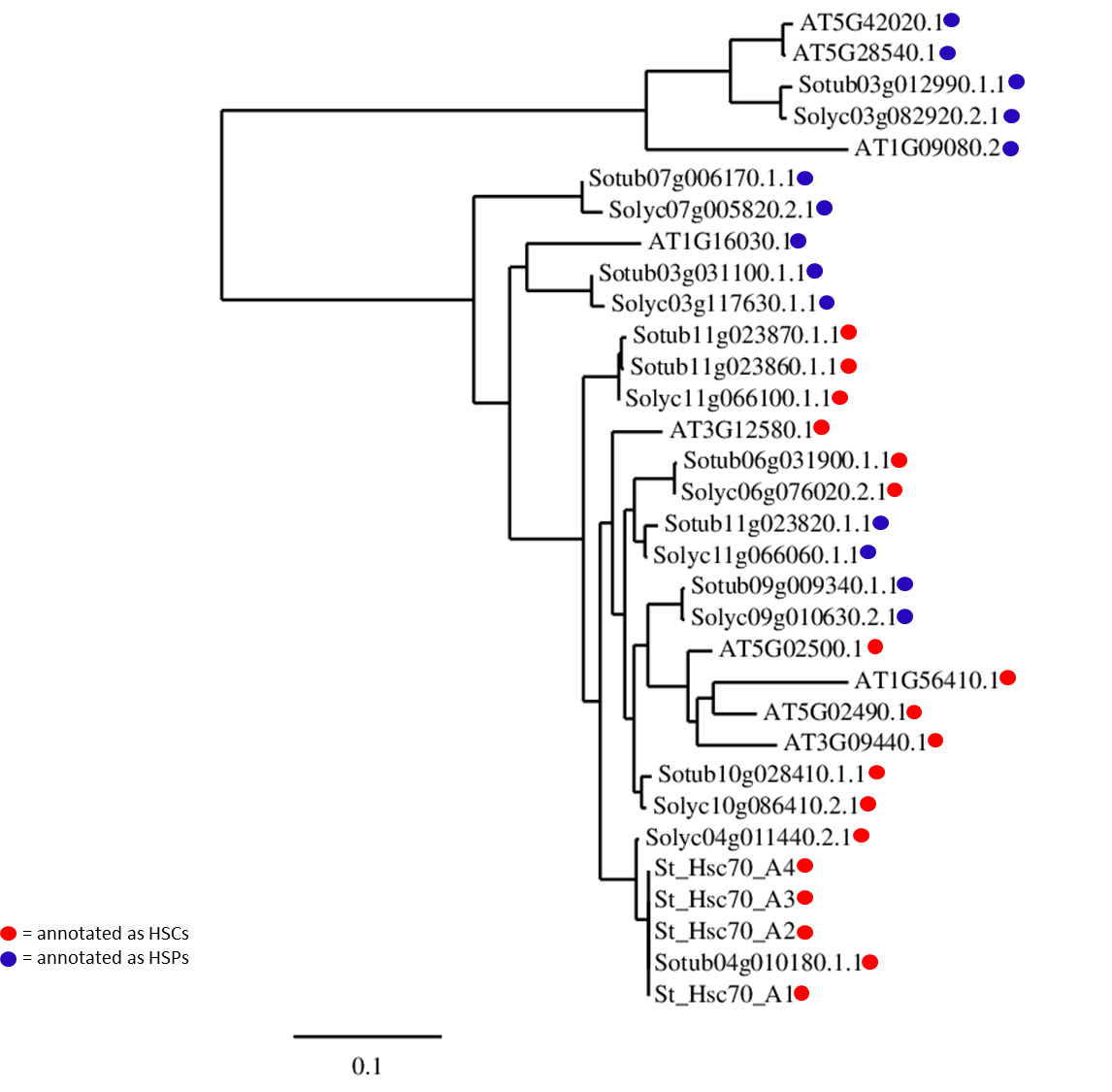


**Figure S5**. Phylogenetic tree of heat shock protein sequences resulting from a BLASTP search against Arabidopsis, Potato and Tomato databases using the translated *HSc70* A2 allele. The tree was generated using Phylogeny.fr web service ([Dereeper et al., 2008](file:///C:\Users\mt40956\AppData\Local\Microsoft\Windows\Temporary%20Internet%20Files\Content.Outlook\87U2KA7R\hsc70_pbt_march_22.docx#_ENREF_10)). The scale bar represents amino acid substitutions per site i.e., the number of changes or 'substitutions' divided by the length of the sequence.
